# Development of an Activity-Based Ratiometric Electrochemical Switch for Direct, Real-Time Sensing of Pantetheinase in Live Cells, Blood, and Urine Samples

**DOI:** 10.1021/acssensors.4c01658

**Published:** 2024-09-27

**Authors:** Namasivayam Kumaragurubaran, Yan-Zhi Huang, Tomas Mockaitis, Ponnusamy Arul, Sheng-Tung Huang, Hsin-Yi Lin, Yi-Cheng Wei, Inga Morkvenaite-Vilkonciene

**Affiliations:** † Department of Chemical Engineering and Biotechnology, National Taipei University of Technology, Taipei 106, Taiwan, ROC; ‡ Institute of Biochemical and Biomedical Engineering, National Taipei University of Technology, Taipei 106, Taiwan, ROC; § Department of Nanotechnology, State Research Institute Centre for Physical Sciences and Technology (FTMC), Sauletekio av. 3, Vilnius 10257, Lithuania; ∥ High-Value Biomaterials Research and Commercialization Center, National Taipei University of Technology, No. 1, Sec. 3, Zhongxiao E. Rd., Taipei 10608, Taiwan, ROC

**Keywords:** biomarkers, latent-probe, ratiometric sensor, pantetheinase, live cells, liquid biopsy

## Abstract

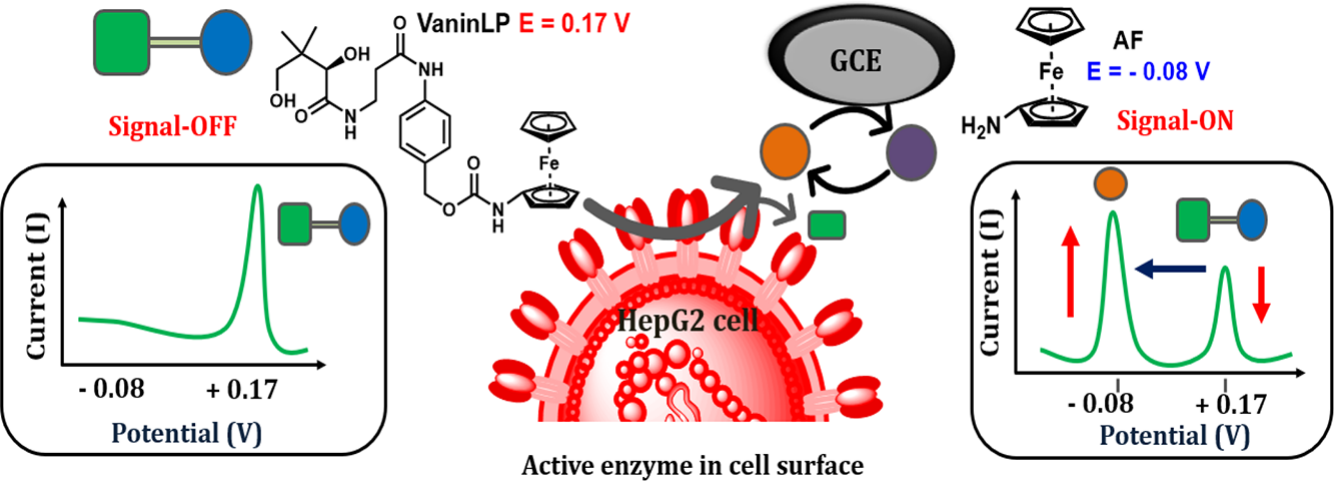

Pantetheinase is a key biomarker for the diagnosis of acute kidney injury and the monitoring of malaria progression. Currently, existing methods for sensing pantetheinase, also known as Vanin-1, show considerable potential but come with certain limitations, including their inability to directly sense analytes in turbid biofluid samples without tedious sample pretreatment. Here, we describe the first activity-based electrochemical probe, termed VaninLP, for convenient and specific direct targeting of pantetheinase activity in turbid liquid biopsy samples. The probe was designed such that cleavage of the pantetheinase amide linkage, triggered by a self-immolative reaction, simultaneously ejects an amino ferrocene reporter. Among the distinctive properties of the VaninLP probe for sensing pantetheinase are its high selectivity, sensitivity, and enzyme affinity, a wide linear concentration range (8–300 ng/mL), and low limit of detection (2.47 ng/mL). The designed probe precisely targeted pantetheinase and was free of interference by other electroactive biological species. We further successfully applied the VaninLP probe to monitor and quantify the activity of pantetheinase on the surfaces of HepG2 tumor cells, blood, and urine samples. Collectively, our findings indicate that VaninLP holds significant promise as a point-of-care tool for diagnosing early-stage kidney injury, as well as monitoring the progression of malaria.

Pantetheinase, commonly known as Vanin-1, is localized via a C-terminal glycosylphosphatidylinositol anchor to the extracellular membrane of epithelial cells,^1−3^ where it hydrolyzes pantetheine to pantothenic acid (vitamin B5) and the amino-thiol cysteamine.^4,5^ Pantetheinase is highly expressed in many organs, including the liver, intestine, and kidney,^2,3^ and plays a crucial role in oxidative stress,^6,7^ pantothenate recycling,^8^ and cell migration.^9^ Clinical functional studies have demonstrated an association between abnormal expression of pantetheinase in various biopsy samples and multiple diseases,^10^ such as diabetes^11^ and influenzas.^12,13^ Recent studies have indicated that pantetheinase activity in urine is a clinically useful diagnostic biomarker of acute kidney injury.^14,15^ In addition, although pantetheinase in blood regulates erythrocyte homeostasis, abnormal levels of pantetheinase in serum are directly associated with a risk for cerebral malaria and severe anemia—the two major manifestations of severe malaria.^16^ Therefore, the development of a convenient method for directly sensing and monitoring the activity of pantetheinase in urine and blood would be a useful tool for acute kidney injury and malaria diagnostics and post-treatment surveillance.

Early efforts to measure the activity of pantetheinase were mainly based on quantification of the reaction product cysteamine through radioactive isotope labeling coupled with paper chromatography, which is technically challenging and prone to inaccuracies.^17^ Other methods, such as enzyme-linked immunosorbent assays (ELISAs)^18^ and activity-based chromogenic methods, exhibit acceptable results, but they also suffer from some drawbacks.^18,19^ As briefly discussed in Table S1, these detection methods depend on specific instruments and entail tedious sample pretreatments and complex assay procedures that require a well-trained technician.^3,18−22^ In modern practice, simple point-of-care detection tools capable of directly assaying actual biopsy samples are essential for rapid on-site diagnosis and post-treatment monitoring. From a clinical perspective, complex liquid biopsy samples, such as blood, are a primary resource for the identification of virtually all disorders.^1,23^ From the perspective of workability, all existing methods for sensing pantetheinase are inconvenient for use as point-of-care tools. Therefore, there is a pressing need for the development of a simple and efficient tool for the direct analysis of the pantetheinase activity in biofluid samples.

Against this backdrop, an activity-based ratiometric electrochemical method that is capable of circumventing limitations of existing detection methods has emerged as an alternative platform for sensing targets in biological systems.^23^ In recent years, our research group has developed various electrochemical ratiometric switches for directly sensing nonredox-active enzyme biomarkers in living cells and blood samples.^24−26^ The developed latent probes are simple, robust molecular switches comprising a masked electrochemical reporter and a targeted analyte-recognition moiety linked to a self-immolative linker.^25,26^ The masked reporter is simultaneously ejected by the target analyte, inducing a chemical reaction with the recognition moiety in the probe molecule that results in a self-immolative conversion that can be observed as a digital signal readout.^24,27^ Moreover, the detection technique exhibits high sensitivity, reliability, and selectivity and shows miniaturization potential; the sensing operating procedure is also simple, requiring only direct mixing of the complex liquid biopsy sample with the latent switches without any tedious sample pretreatment procedures. A key advantage of this method compared with chromogenic detection^19−22^ is its ability to directly convert the analyte–probe interaction into a digital signal without any additional devices. Given their tremendous advancements,^24−28^ our latent electrochemical detection methods hold potential for future development as point-of-care detection kits.

In this study, we designed and developed the first self-immolative ratiometric electrochemical latent probe, termed VaninLP, for analyzing the activity of pantetheinase in live cells and real biofluid samples. The chemical structure of the latent probe consists of an amino ferrocene derivative (AF) reporter unit linked to an amino benzyl alcohol self-immolative linker, which is coupled to a pantothenic acid trigger via an amide linkage. Because of amino ferrocene’s linkage to an electron-withdrawing carbamate functional group, the VaninLP latent probe displays a redox signal in the positive voltage region (+0.17 V vs Ag/AgCl) in voltammograms. Cleavage of the pantetheinase amide linkage triggers a self-immolative reaction that simultaneously ejects the electron-rich AF reporter, which “turns on” its redox activity in the negative potential region (−0.08 V vs Ag/AgCl).^29^ The resulting VaninLP probe displayed a linear relationship between the signal and the concentration of pantetheinase over a wide dynamic range, was highly specific and sensitive, and showed excellent stability. Last, the VaninLP probe was able to successfully detect and quantify pantetheinase activity in blood and urine samples, as well as on the surface of tumor cells, all without time-consuming sample processing. To the best of our knowledge, this is the first reported electrochemical detection method for pantetheinase.

## Experimental Section

### Materials and Methods

Detailed descriptions of the materials and procedures used to synthesize the probe are given in the Supporting Information (SI. 1). The preparation of stock solutions, optimization studies, cell-culture methods, and assaying of biofluids (blood and urine) are given in the Supporting Information (SI. 2–5).

### Assaying the Activity of Pantetheinase Using the VaninLP Probe

An aqueous stock solution (5 mM) of latent probes was prepared by dissolving in HEPES (2-[4-{2-hydroxyethyl}piperazin-1-yl]ethanesulfonic acid) and combining with dimethyl sulfoxide (DMSO) at a 50:50 (volume/volume) ratio. Aliquots (1 mL) of pantetheinase test samples at different concentrations (8–300 ng/mL), prepared from a 1 μg/mL stock, were mixed with a 0.1 M HEPES buffer (pH 7.0) solution containing VaninLP probe (50 μM stock in 50:50 (volume/volume) DMSO:HEPES) and 50 μM 1,4-dithiothretitol (DTT). The assay mixture was incubated for 30 min at 37 °C and then transferred to an electrochemical cell for analysis of released AF using differential pulse voltammetry (DPV).

### Detecting Cellular Pantetheinase Activity on the Surface of Tumor Cells

To monitor and measure the production of pantetheinase within cells, we cultured HepG2 cells (1 × 10^6^ cells/mL) directly in electrochemically working cells. We then removed the medium and replaced it with 1 mL of a 0.1 M HEPES buffer (pH 7.0) solution containing 50 μM VaninLP probe and 100 μM DTT, followed by incubation for 5 h at 37 °C. Thereafter, the electrochemical cell was transferred to an electrochemical workstation, and pantetheinase activity on the cell surface was analyzed by DPV.

### Evaluating Pantetheinase in Newborn Bovine Calf Serum (NBCS) Samples

Assays (total volume, 1 mL) were prepared by adding 50 μL of NBCS to a 0.1 M HEPES (pH 7.0) solution containing 50 μM VaninLP probe and 100 μM DTT. The prepared assay was incubated at 37 °C for 30 min and then transferred to an electrochemical cell for measurement of pantetheinase activity by DPV.

### Evaluating Pantetheinase in Blood Samples

Assays (total volume, 1 mL) were prepared by adding 500 μL of blood sample to a 0.1 M HEPES (pH 7.0) solution containing 50 μM VaninLP probe (5 mM stock in 50:50 (volume/volume) DMSO:HEPES buffer) and 50 μM DTT. The prepared test samples were incubated for 30 min at 37 °C and then transferred to an electrochemical cell for measurement of pantetheinase activity by DPV.

### Evaluating Pantetheinase in Urine Samples

Assays (total volume, 1 mL) were prepared by adding 200 μL of urine sample to a 0.1 M HEPES (pH 7.0) solution containing 50 μM VaninLP probe (5 mM stock in 50:50 (volume/volume) DMSO:HEPES buffer) and 50 μM DTT. The prepared test samples were incubated for 30 min at room temperature and then transferred to an electrochemical cell for measurement of pantetheinase activity by DPV.

### Real-Time Sensing of Pantetheinase and Its Inhibition in Blood and NBCS Samples

To quantify and monitor pantetheinase activity in blood samples in real time, we prepared assays (total volume, 1 mL) by adding 500 μL of blood to a 0.1 M HEPES (pH 7.0) solution containing 50 μM VaninLP probe (5 mM stock in 50:50 (volume/volume) DMSO:HEPES buffer) and 50 μM DTT. Analyses were performed with and without 1 μM RR6 (0.05 mM stock in HEPES), a pantetheinase inhibitor, added either at the start of the reaction or after 30 min. Prepared samples were immediately transferred to an electrochemical cell for the real-time analysis of pantetheinase activity by DPV. Real-time analysis of pantetheinase activity in NBCS was performed following the same protocol. Specifically, assays (total volume, 1 mL) were prepared by adding 50 μL of NBCS to a 0.1 M HEPES (pH 7.0) solution containing 50 μM VaninLP probe (5 mM stock in 50:50 (volume/volume) DMSO:HEPES buffer) and 50 μM DTT. Assays were performed with and without 1 μM RR6 as described for blood, and samples were immediately transferred to an electrochemical cell for real-time analysis of pantetheinase activity by DPV.

### Real-Time Sensing of Pantetheinase and Its Inhibition in Urine Samples

To quantify and track pantetheinase activity in real-time in urine samples, we prepared a 1 mL assay mixture containing 200 μL of urine sample, 50 μM VaninLP probe (5 mM stock in 50:50 (volume/volume) DMSO:HEPES buffer), 0.1 M HEPES (pH 7.0), and 50 μM DTT, spiked with 100 ng/mL pantetheinase. The prepared samples were immediately added to the electrochemical cell for DPV analysis, which yielded pantetheinase activity at regular 10 min intervals for a duration of 100 min. Assays were performed with and without 1 μM RR6 (0.05 mM stock in HEPES), added either at the start of the reaction or after 30 min.

## Results and Discussion

### Design and Synthesis of VaninLP

The synthesis of the target VaninLP probe was performed in four steps from the known starting synthon (Scheme S1). The detailed description of the synthetic procedures and the characterization of chemical structures of synthetic intermediates and VaninLP are described in detail in the Supporting Information (SI. 1.1–1.4). The chemical structures of VaninLP and their synthetic intermediates were confirmed by NMR, FT-IR, and ESI mass spectrometry, and they are presented in Figures S1–S8.

### Evaluating the Electrochemical Signal Performances of VaninLP Probe for Sensing Pantetheinase Activity

**Scheme 1:**
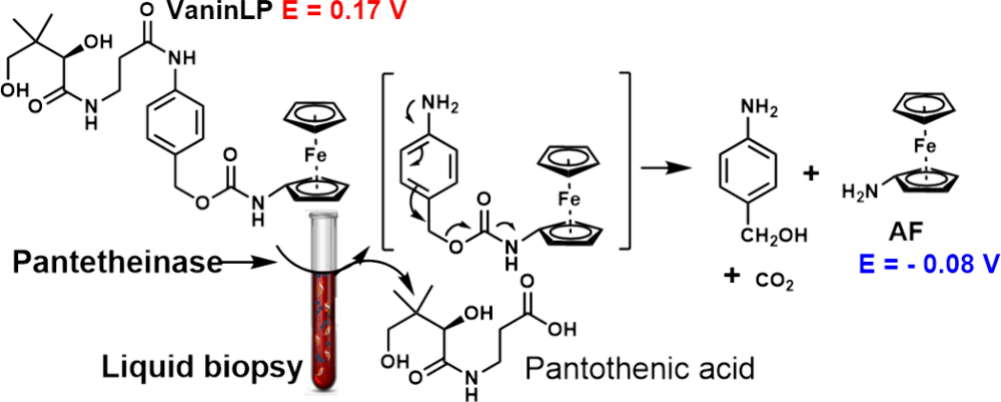
Schematic Showing the Working Principle of the Activity-Based Probe, VaninLP, for the Detection of Pantetheinase

**Figure 1:**
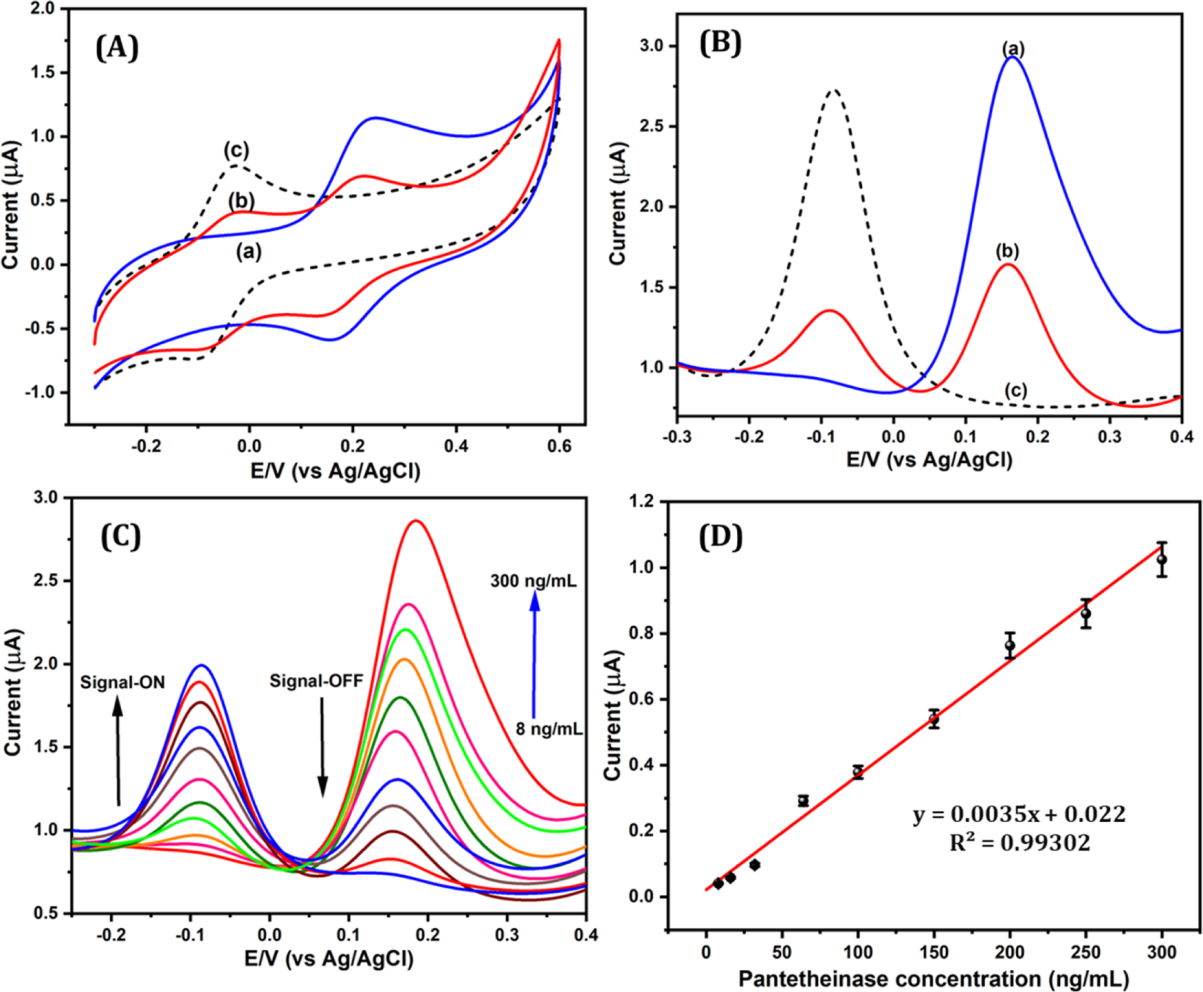
(A) CV and (B) DPV of 50 μM VaninLP in the absence (curve a) and presence (curve b) of 120 ng/mL pantetheinase and 50 μM pristine AF (curve c). (C) DPV of 50 μM VaninLP with concentrations of pantetheinase ranging from 8 to 300 ng/mL, whose individual concentrations are 8, 16, 32, 64, 80, 100, 150, 200, 250, 280, and 300 ng/mL. (D) Calibration plot of VaninLP current (AF signal) responses versus concentration of pantetheinase (ng/mL). All samples were analyzed in DMSO:HEPES buffer (pH = 7.0; 37 °C).

We began by investigating the latent VaninLP signal, revealing the reaction triggered by pantetheinase using two different types of voltammetric detection techniques. Cyclic voltammetry (CV) was used to characterize the electrochemical redox profile of VaninLP with and without pantetheinase (Figure 1A). The latent probe, VaninLP, displayed a sharp redox couple at a standard potential (*E*_o_′) of +0.17 V (Figure 1A, curve a). Co-incubation of VaninLP with pantetheinase gave rise to a new redox couple at −0.08 V that exhibited a significant decrease in current at a positive peak potential (*E*_o_′) of +0.17 V (Figure 1A, curve b). The redox potential of the newly formed redox couple was similar to that of the pristine signal response of the AF reporter (*E*_o_′ = −0.08 V) (Figure 1A, curve c).^28^ Thus, the switch of the redox couple can be attributed to a reversible one-electron transfer between ferrocene and ferrocenium cation.^23−26^ Detailed optimization of the working conditions for sensing, including pH, percentage of DMSO, probe concentrations, and incubation time, is presented in the Supporting Information (Figure S9A–C). Next, differential pulse voltammetry (DPV) was employed to investigate the electrochemical signal performance of VaninLP in the presence of pantetheinase (Figure 1B). In the absence of pantetheinase, VaninLP exhibited an oxidation peak at +0.17 V (Figure 1B, curve a). Co-incubation of VaninLP with pantetheinase significantly decreased the current response of VaninLP at +0.17 V and resulted in the appearance of a new oxidation current response at a negative peak potential of −0.08 V (Figure 1B, curve b). The newly arising oxidation peak potential matched that of the signals of the AF reporter and validated the cyclic voltammetric peak signal (Figure 1B, curve c).^25,26^ Because the electron-rich species exhibited lower potentials, these results are consistent with pantetheinase catalyzing the self-immolative reaction of VaninLP to release the electron-rich AF (Scheme 1 and Scheme S2).

To determine the sensing capacity of pantetheinase, we incubated 50 μM VaninLP with different concentrations of pantetheinase under optimized conditions and monitored the resulting current responses by DPV (Figure 1C). The anodic peak current (*I*_pa_) of AF increased linearly with increases in pantetheinase concentration (8 to 300 ng/mL), and the current responses of VaninLP decreased concomitantly. The concentration of pantetheinase was correlated with *I*_pa_ of AF. The calibration plot of *I*_pa_ at −0.08 V against the concentration of pantetheinase was found to be linear across a range from 8 to 300 ng/mL (Figure 1D). The limit of detection (LOD), calculated using the expression 3σ/slope, was determined to be 2.47 ng/mL. To the best of our knowledge, this is the first electrochemical sensing method for determining pantetheinase activity with a direct digital readout. Additionally, the analytical performance of the VaninLP probe was found to be equivalent to or better than recently reported colorimetric and fluorescent pantetheinase detection methods, with published LODs of 1.5 × 10^–4^ U/mg, 4.7 ng/mL, 0.37 ng/mL, and 0.69 ng/mL, respectively (Table S1). Thus, our VaninLP probe has the capacity to quantitatively track the pantetheinase activity with a direct digital readout. It also holds the potential for miniaturization for future expansion toward a future point-of-care diagnostic tool.

### Selectivity and Kinetics of the VaninLP Probe toward Pantetheinase

We next evaluated the selectivity of the VaninLP probe, testing its ability to undergo the self-immolative reaction in response to other potential interfering species commonly found in liquid biopsy specimens, such as 0.5 mM concentrations of inorganic salts (copper chloride, magnesium chloride, ferrous chloride, and calcium chloride), ascorbic acid, dopamine, uric acid, l-cysteine, glutathione, glucose, creatine, urea, calcium pantothenate, H_2_O_2_, amino acids (valine, phenylalanine, tryptophan, asparagine, threonine, sarcosine, alanine, lysine, glycine, proline, and arginine), and 10 U/L protease enzymes (GGT, esterase, leucine aminopeptidase, dipeptyl peptidase (DPP-IV), trypsin, and aminopeptidase-N (APN)); those DPV graphs are shown in Figures S10A and S11. No negative current responses (−0.08 V vs Ag/AgCl) were observed upon incubation of VaninLP in the presence of any of the above-mentioned species (Figure S10A), confirming that current responses at −0.08 V could only be induced by incubation of VaninLP with pantetheinase. We further investigated the affinity of VaninLP for pantetheinase through enzyme kinetic experiments (Figure S10B–D). The *K*_m_ and apparent *V*_max_ of VaninLP against pantetheinase calculated from these experiments were 2.38 μM and 1.25 nM/min/ng, respectively. The *K*_m_ and apparent *V*_max_ values of VaninLP against pantetheinase were similar to or better than (i.e., lower than) those for most reported activity-based fluorescent probes that have been published with *K*_m_ values of 0.937 and 3.356 μM and apparent *V*_max_ values of 120 and 192.5 nM/min/ng, respectively, and typical substances, as shown in Table S1. Collectively, these results indicate that the VaninLP probe has strong selectivity toward pantetheinase relative to other interfering species, as shown in Figure S10A.

### Quantitative Analysis of the Pantetheinase Activity of Tumor Cell Surfaces

**Figure 2:**
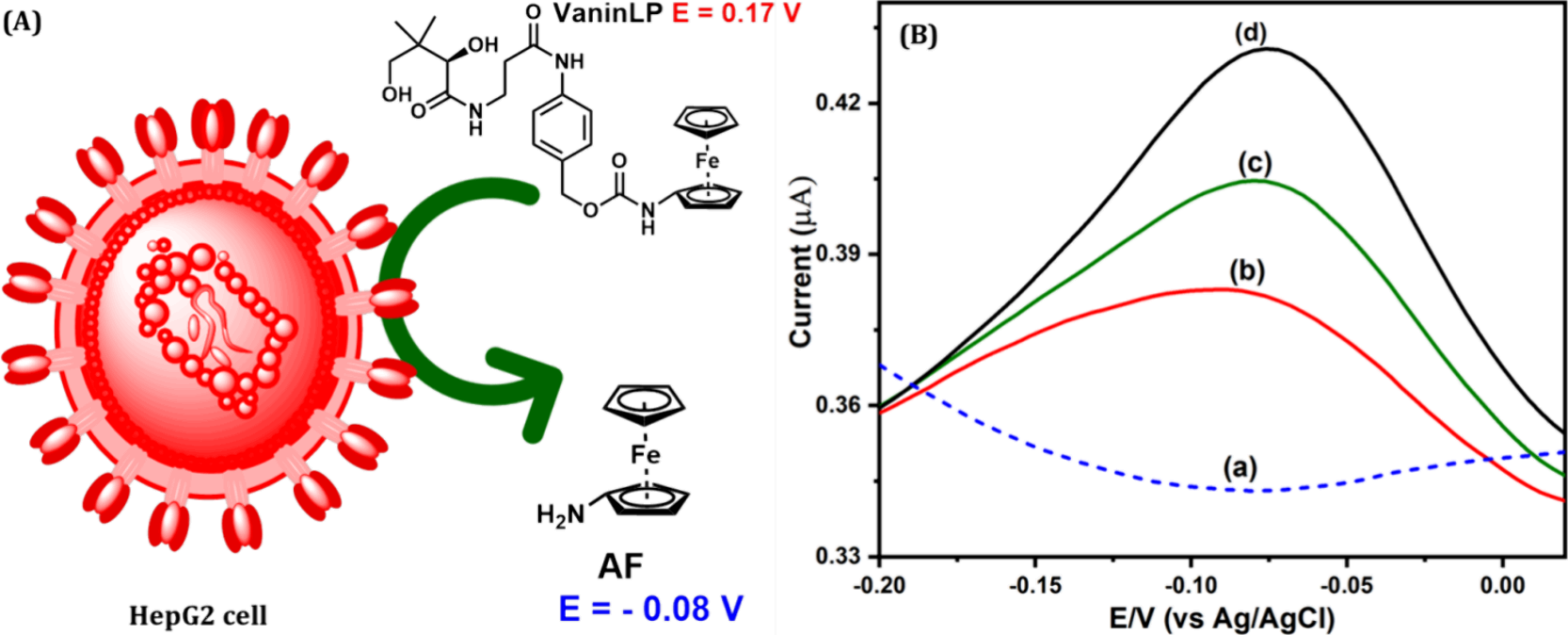
(A) Schematic illustration of VaninLP profiling of pantetheinase in HepG2 cells. (B) DPV curves of HepG2 cells alone (curve a), VaninLP incubated with HepG2 cells (curve b), and HepG2 incubated with the probe and indicated amounts of pantetheinase (curves c and d).

Pantetheinase is an ectoenzyme commonly expressed on various tumor cell surfaces; it is also a useful biomarker for the diagnosis of kidney injury diseases and has been linked to epithelial barrier damage and proliferation and spread of tumor cells.^29,30^ Therefore, determining the effectiveness of a tumor treatment would benefit from the ability to quantify the pantetheinase activity. Initially, we used the MTT (3-[4,5-dimethylthiazol-2-yl]-2,5 diphenyl tetrazolium bromide) assay to evaluate the cytotoxicity of VaninLP, specifically employing HepG2 cells cultured using comparable protocols (Supporting Information, SI. 2). As illustrated in Figure S12, more than 94.0% of originally live HepG2 cells remained viable after treatment with 50 μM VaninLP for 6 h (*n* = 3 experiments). These results suggest that VaninLP can be successfully applied to practical sample analysis of live cells without causing overt toxicity. We then investigated the electrochemical signal response of pantetheinase activity in live cells by DPV. At a negative potential region (−0.08 V vs Ag/AgCl) of the electrochemical spectrum, no notable current response was produced during incubation of HepG2 cells alone for 5 h at 37 °C (Figure 2B, curve a). In contrast, co-incubation of the VaninLP probe with HepG2 cells for 5 h at 37 °C under optimized conditions produced a current response at a negative potential of −0.08 V (Figure 2B, curve b). Quantification of these results, displayed in Table 1, yielded a value of 3.36 ng/mL pantetheinase, as estimated from the calibration plot (Figure S13). We next quantitatively measured the amount of pantetheinase activity released in cells after spiking assay mixtures containing HepG2 cells with 1 or 2 ng/mL pantetheinase. We observed an enhanced electrochemical response in each case, with pantetheinase activity determined to be 4.32 and 5.34 ng/mL, respectively (Figure 2B, curves c and d). The spiked-recovery results were in good agreement, with the measured activity values falling within a 2.0% error of the spiked pantetheinase quantity. From this, we conclude that VaninLP is a useful molecular probe for identifying pantetheinase activity directly in living cells, as shown in the scheme depicted in Figure 2A. Our platform also demonstrated the capacity to directly quantify pantetheinase on the cell surface. Since pantetheinase has been found to be an important target for the creation of anticancer medications, our method, which involves detecting pantetheinase activity immediately when it is impacted by an antagonist, might prove to be a useful tool for the future development of anticancer medications.

**Table 1:** Amount of Pantetheinase Measured in HepG2 Cell Samples Spiked with the Indicated Amount of Pantetheinase

**real sample**	**spiked pantetheinase** (ng/mL)	**measured current (μA)**	**measured pantetheinase** (ng/mL)^a^	**percent error (%)**^b^
cell: probe + HEPES	—	0.039 ± 0.02	3.36 ± 0.05	—
cell: probe + HEPES + pantetheinase spike-1	1.00	0.048 ± 0.12	4.34 ± 0.04	2
cell: probe + HEPES + pantetheinase spike-2	2.00	0.057 ± 0.04	5.32 ± 0.03	2

a Reported pantetheinase values were adjusted according to the dilution factor to reflect pantetheinase values in HepG2 cell samples.

b Percent error (%) = measured pantetheinase value with spike (observed) – pantetheinase value with spike (theoretical) × 100 pantetheinase spike value.

### Direct Real-Time Quantification of Pantetheinase Activity in NBCS

**Figure 3:**
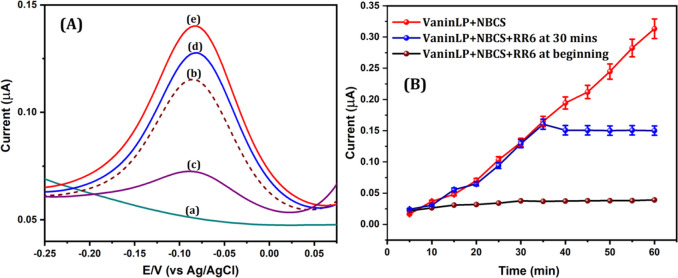
(A) DPV of NBCS alone (curve a), VaninLP probe incubated with NBCS (curve b), NBCS incubated with the probe and RR6 (curve c), and NBCS incubated with the probe and the indicated amounts of pantetheinase (curves d and e). (B) Real-time detection. Plot of current response versus time, measured every 5 min for 60 min, without RR6 and with RR6 added at the beginning or after 30 min.

The activity of pantetheinase in serum samples has been previously reported.^21^ Based on the importance of biofluid samples and considering the demonstrated interference-free capacitance of VaninLP, we extended our pantetheinase monitoring method to the quantitative determination of pantetheinase activity directly in real NBCS samples. NBCS initially incubated alone for 30 min at 37 °C under optimized conditions produced no significant response current in the negative potential region (−0.08 V vs Ag/AgCl), as shown in Figure 3A (curve a). After co-incubation of NBCS with VaninLP at 37 °C for 30 min (Figure 3A, curve b), a specific current response that corresponded to 3.2 μg/mL pantetheinase, as estimated from the standard calibration plot, was obtained (Figure 1D), an amount similar to that estimated using a fluorescence-based method.^3^ Co-incubation of NBCS and VaninLP with 100 μM RR6, a pantetheinase analogue and selective inhibitor, resulted in a diminished electrochemical signal response (Figure 3A, curve c). In addition to confirming the specificity of the signal, the finding of effective inhibition by RR6 establishes VaninLP as a suitable molecular probe for identifying pantetheinase inhibitors in serum samples. NBCS and VaninLP co-incubated together with spiking of 0.6 and 1.2 μg/mL pantetheinase likewise showed an enhanced current signal response at −0.08 V (Figure 3A, curves d and e). Corresponding pantetheinase activity levels, estimated from a standard calibration curve, were determined to be 3.82 and 4.45 μg/mL (Table 2), values that were in good agreement with spiked pantetheinase quantity (within a 4.0% error).

**Table 2:** Amount of Pantetheinase Measured in NBCS Spiked with the Indicated Amount of Pantetheinase

**real sample**	**spiked pantetheinase** (μg/mL)	**measured current (μA)**	**measured pantetheinase** (μg/mL)^a^	**percent error (%)**^b^
NBCS: probe + HEPES	—	0.582 ± 0.02	3.20 ± 0.03	—
NBCS: probe + HEPES + pantetheinase spike-1	0.6	0.690 ± 0.03	3.82 ± 0.06	2.87
NBCS: probe + HEPES + pantetheinase spike-2	1.2	0.800 ± 0.01	4.45 ± 0.22	3.83

a Reported pantetheinase values were adjusted according to the dilution factor to reflect pantetheinase values in NBCS samples.

b Percent error (%) = measured pantetheinase value with spike (observed) – pantetheinase value with spike (theoretical) × 100 pantetheinase spike value.

We also examined the ability of our probe to monitor pantetheinase activity in real time. We carried out three distinct experiments (collectively displayed in Figure 3B): (i) co-incubation of VaninLP with NBCS at room temperature under optimal conditions, with real-time (every 5 min for 60 min) monitoring of pantetheinase activity by DPV (Figure 3B, red curve; individual DPV curves are displayed in Figure S14); (ii) co-incubation of VaninLP with NBCS at room temperature under optimal conditions in the presence of the inhibitor, RR6, added at the beginning of the reaction; and (iii) same as (ii) but with RR6 added at the 30 min mark. In experiment ii, there was no discernible current response from beginning to end (Figure 3B, black curve); in experiment iii, the current response was diminished rather than increased after adding RR6 (Figure 3B, blue curve). The designed latent probe demonstrated the capacity to measure pantetheinase activity directly in serum samples. Employing the VaninLP probe in a serum sample, we also confirmed that our platform has the ability to monitor the activity of pantetheinase in real time. The development of pantetheinase-specific inhibitors has garnered significant attention in pharmacological research owing to its potential as a biomarker for the early identification of renal damage.^30,31^ Considering these properties, the VaninLP probe may serve as a good tool for examining the efficiency of the inhibitor.

### Direct Real-Time Quantification of Pantetheinase Activity in Blood and Urine Samples

**Figure 4:**
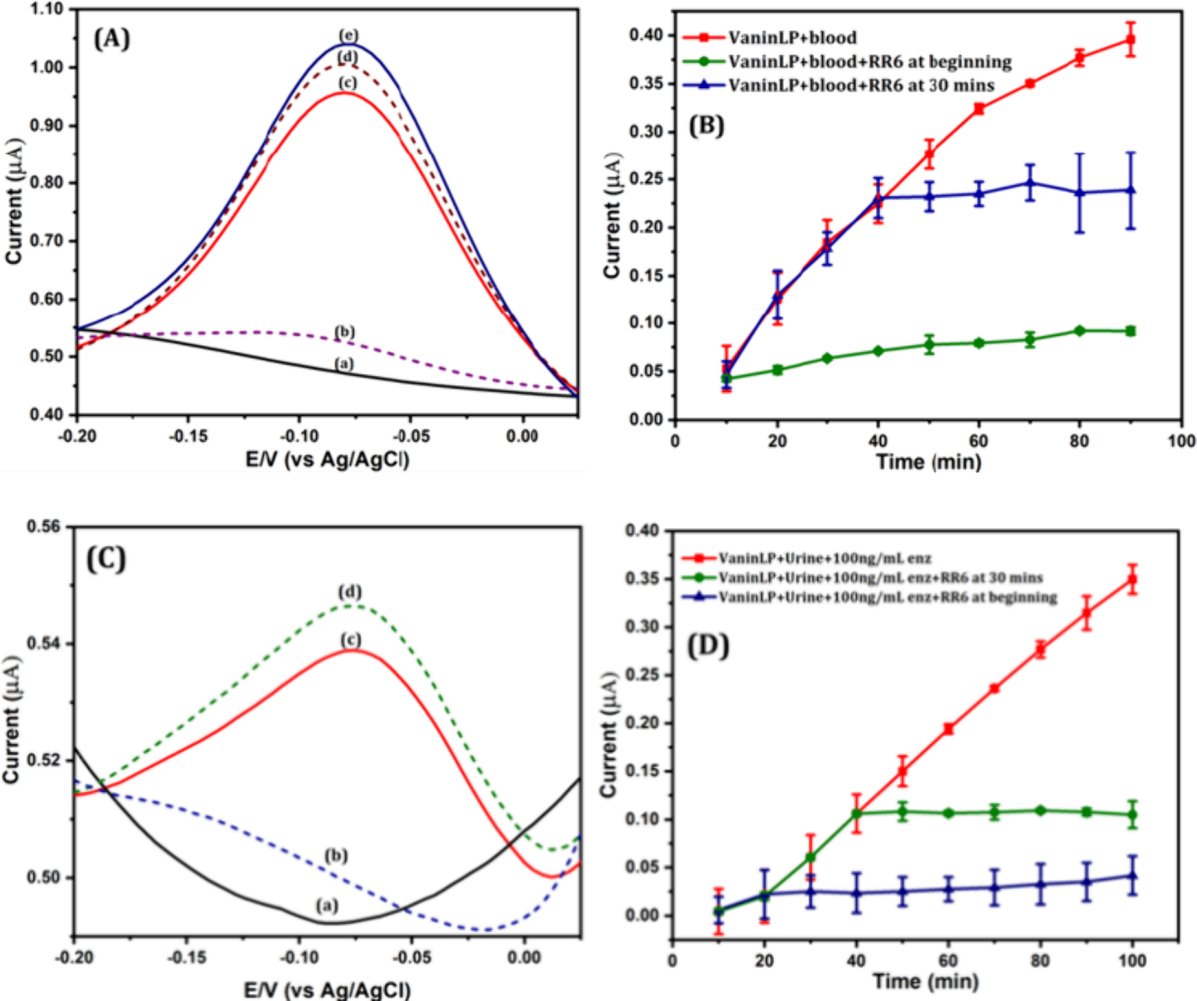
(A) DPV of blood alone (curve a), VaninLP incubated with blood (curve b), VaninLP incubated with blood and RR6 (curve c), and VaninLP incubated with blood and different amounts of spiked pantetheinase (curves d and e) of 30 and 36 ng/mL, respectively. (B) Real-time detection. Plot of current versus time for blood without RR6 and with RR6 added at the beginning or at 30 min. Values were measured every 10 min for 90 min. (C) DPV of urine alone (curve a), VaninLP incubated with urine (curve b), and VaninLP incubated with urine and the indicated amounts of spiked pantetheinase (curves c and d) of 50 and 55 ng/mL, respectively. (D) Real-time detection. Plot of current versus time for urine samples without RR6 and with RR6 added at the beginning or at 30 min. Values were measured every 10 min for 100 min.

Clinically abnormal levels of pantetheinase in the blood and urine are directly associated with human health problems. Furthermore, patients with bladder or pancreatic cancer have higher levels of circulating pantetheinase in their plasma and urine compared with healthy controls.^32,33^ It has also been proposed that pantetheinase activity in the blood and urine of high-risk patients should be tracked to diagnose and monitor acute kidney injury. Accordingly, we examined the ability of our latent electrochemical probe, VaninLP, to detect pantetheinase activity directly in the blood and urine with minimal pretreatment. Under optimized conditions, a DPV analysis of 50% (volume/volume) blood incubated alone at 37 °C for 30 min produced no current signal in the negative potential region (Figure 4A, curve a). In contrast, VaninLP directly co-incubated with blood produced a significant current response similar to that of the pristine AF response current (Figure 4A, curve b) that corresponded to a value of 314.2 ng/mL pantetheinase, as estimated from the calibration plot (Figure 1D). Additionally, the electrochemical current response to co-incubation of VaninLP with blood was suppressed in the presence of RR6 (Figure 4A, curve c). In addition, spiking co-incubations of VaninLP and blood with 30 and 36 ng/mL pantetheinase produced a noticeably stronger signal current. Corresponding pantetheinase values obtained by reference to a standard curve were 344.6, and 349.8 ng/mL, respectively (Table 3), with the measured activity values falling within a 5.0% error of the spiked pantetheinase quantity. Several reports have indicated that serum pantetheinase activity likely represents GPI-80, which is known to be present in its secreted form. Serum levels of GPI-80 in healthy individuals have been reported to be ∼100 ng/mL^34^; however, there have been no reports on the amount of pantetheinase in blood, which includes the surface of erythrocytes. Having successfully demonstrated that our detection method is able to directly detect and quantify pantetheinase in blood without tedious serum/plasma separation procedures, we are currently collecting more blood samples to validate our testing methods against other existing methods.

**Table 3:** Amount of Pantetheinase Measured in Blood and Urine Samples Spiked with the Indicated Amount of Pantetheinase

**real sample**	**spiked pantetheinase** (ng/mL)	**measured current (μA)**	**measured pantetheinase** (ng/mL)^a^	**percent error (%)**^b^
Blood				
blood: probe + HEPES (1:1 (volume/volume)) dilution	—	0.572 ± 0.03	314.2 ± 0.13	—
blood: probe + HEPES + pantetheinase spike-1 (1:1)	30.00	0.625 ± 0.02	344.6 ± 0.31	1.33
blood: probe + HEPES + pantetheinase spike-2 (1:1)	36.00	0.634 ± 0.11	349.8 ± 0.30	1.11
Urine				
urine: probe + HEPES (1:5 (volume/volume)) dilution	—	—	—	—
urine: probe + HEPES + pantetheinase spike-1 (1:5)	50.00	0.035 ± 0.04	48.55 ± 0.02	2.90
urine: probe + HEPES + pantetheinase spike-2 (1:5)	55.00	0.039 ± 0.03	54.3 ± 0.03	1.27

a Reported pantetheinase values were adjusted according to the dilution factor to reflect pantetheinase values in blood and urine samples.

b Percent error (%) = measured pantetheinase value with spike (observed) – pantetheinase value with spike (theoretical) × 100 pantetheinase spike value.

We also demonstrated the capacitance of pantetheinase activity in 20% (volume/volume) urine samples. Neither urine alone nor urine co-incubated with VaninLP produced a sizable response current (Figure 4C, curves a and b). Generally, very little or no pantetheinase is present in urine, with an exception being terminally ill patients. It has been reported that the median urinary pantetheinase level (interquartile range) is 0.33 (0–2.6) ng/mg Cr.^35^ Here, we found that spiking 50 or 55 ng/mL pantetheinase into VaninLP co-incubated with urine resulted in a notable current in the negative region (Figure 4C, curves c–e). As shown in Table 3, the calculated amounts of pantetheinase, as estimated from the calibration curve, were 48.55 and 54.3 ng/mL, respectively (Figure S15). These findings demonstrate the reliability of the standard spectral signal and the capacity of VaninLP to directly quantify pantetheinase activity in turbid samples, including blood and urine, without alterations in the detection peak potential.

Interestingly, our developed platform using VaninLP also showed the ability to track the activity of pantetheinase in real time in blood and urine samples. We performed real-time monitoring studies on blood, co-incubating VaninLP with 50% blood samples under optimal conditions and recording DPV every 10 min (Figure 4B, red curve). Individual DPV curves are displayed in Figure S16. We also co-incubated VaninLP and 50% blood with the pantetheinase inhibitor RR6, added at different times. When added at the beginning of the assay (Figure 4B, green curve), RR6 prevented the development of a discernible current response throughout the monitoring period. Following addition of RR6 at the 30 min mark, the current response was decreased rather than increased (Figure 4B, blue curve). We also performed similar real-time tracking experiments on urine samples with and without added RR6 at the beginning of the assay and at 30 min. Since we knew that, under ideal conditions, the amount of pantetheinase in urine is very low, we added 100 ng/mL of the enzyme and made DPV recordings every 10 min (Figure 4D, red curve). Individual DPV curves are displayed in Figure S17. As was the similar case like blood experiments, co-incubation of VaninLP, 20% urine, and 100 ng/mL pantetheinase did not produce a current throughout the recording period when RR6 was added at the beginning of the assay (Figure 4D, blue curve) and decreased rather than increased when RR6 was added at the 30 min mark (Figure 4D, green curve).

It is also known that abnormal levels of pantetheinase in the urine or blood are indicative of kidney damage or correlate with the severity of malaria. According to the World Health Organization (WHO), the African region accounted for 95% of malaria cases and 96% of fatalities worldwide in 2021, during which there were 247 million cases and 619,000 deaths from malaria. In addition, 80% of malaria deaths were in children under the age of five.^36^ Medical examination rooms are scarce in these countries, highlighting the importance of simple and convenient tools for pre- and post-treatment monitoring. Currently available chromogenic reagents for detecting pantetheinase can only be used with serum, and sample pretreatment is required prior to their use with sensing reagents. Thus, additional equipment is needed for the sensing procedure, and the results are typically delayed. In contrast to conventional methods, our ratiometric probe method is designed to allow direct mixing of blood samples with the probe, requiring fewer steps involving the handling of liquid samples. Importantly, from the standpoint of controlling kidney injury and malaria, determining pantetheinase concentrations can assist with medication dosage decisions, intervention effectiveness assessments, and recurrence monitoring. Therefore, our platform would be an effective tool for measuring the kidney-injury biomarker, pantetheinase. It would also be relevant for future deployment for point-of-care and post-treatment surveillance of subsequent recurrence of cancer, given its quantitative output compared with basic analytical procedures.

## Conclusions

In summary, we constructed the first activity-based ratiometric electrochemical probe designed to measure pantetheinase activity in turbid biopsy samples. The developed probe, VaninLP, was found to demonstrate excellent electrochemical properties, including strong affinity (*K*_m_) and a broad dynamic concentration range with commendable detection sensitivity and specificity. We further confirmed the analytical performance of VaninLP with minimal equipment and a few liquid handling steps by measuring pantetheinase activity in blood and urine and on the surfaces of tumor cells (HepG2). We showed that our developed electrochemical sensing platform could be a useful tool in clinical practice with the potential to provide an early diagnosis of pantetheinase activity in tumor cells. We are currently investigating ways to further improve the method to make it easier for end users, including efforts to accommodate a decreased sample volume and permit the use of disposable electrode strips.

## Data Availability

Data will be made available on request.

## Supporting Information Available

Additional experimental and characterization details (PDF)
