# Supplementary material for: Development of an Activity-Based Ratiometric Electrochemical Switch for Direct, Real-Time Sensing of Pantetheinase in Live Cells, Blood, and Urine Samples
Source: ACS Sens. 2024 Sep 27;9(10):5436–44. doi: 10.1021/acssensors.4c01658 (PMC11519916; doi:10.1021/acssensors.4c01658)
Supplement: Supplementary file 1 — se4c01658_si_001.pdf [file se4c01658_si_001.pdf]

Supporting information

Development of an Activity-Based Ratiometric Electrochemical Switch for Direct, Real-Time Sensing of Pantetheinase in Live Cells, Blood, and Urine Samples

Namasivayam Kumaragurubaran [a,b], **Yan-Zhi Huang** [a,b], **Tomas Mockaitis** [c], **Ponnusamy Arul** [a,b], **Sheng-Tung Huang** [a,b,d]*, **Hsin-Yi Lin** [a], **Yi-Cheng Wei** [a], **Inga Morkvenaite-Vilkonciene** [c]*

[a]Department of Chemical Engineering and Biotechnology, National Taipei University of Technology, Taipei 106, Taiwan, ROC

[b]Institute of Biochemical and Biomedical Engineering, National Taipei University of Technology, Taipei 106, Taiwan, ROC

[c]Department of Nanotechnology, State Research Institute Centre for Physical Sciences and Technology (FTMC), Sauletekio av. 3, 10257 Vilnius, Lithuania

[d]High-Value Biomaterials Research and Commercialization Center, National Taipei University of Technology, No. 1, Sec. 3, Zhongxiao E. Rd., 10608, Taipei, Taiwan, ROC

Corresponding authors:

E-mail: ws75624@ntut.edu.tw (S.T. Huang); inga.vilkonciene@ftmc.lt (I. Morkvenaite-Vilkonciene).

Table S1: Comparison of the performances of VaninLP and other analytical tools for assaying pantetheinase activity.

Method	Assay time	Merits	Limitations	LOD	Real sample	K_m	V_{max}	Reference
**ELISA**	1hr	Highly sensitive, specific, and reproducible	Time-consuming since it needs specialised chemicals, a specialised apparatus to transform the signal, and multiple washing procedures.	-	Serum and urine	-	-	(1)
**Colorimetry**	30 min	Extremely easy to use, economical, and sensitive	Lack of reusablity, other interferences include bubbles in the sample cells or tubes, turbidity of the sample, and sample color	1.5×10^{-4} U/mg	-	28 µM	-	(2)
**Fluorescence-1 CV-PA** 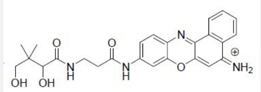	60 min	Both in vitro and in vivo imaging, highly sensitive and selective.	Not suitable for turbid samples and necessitates the use of an additional device to convert the optical signal to a digital signal.	4.7 ng/mL	Blood serum	78 µM	-	(3)
**Fluorescence-2 TMN-PA** 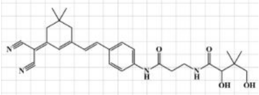	60 min			0.37 ng/mL	Cells and in vivo imaging	0.937 µM	120 nM/min /ng	(4)
**Fluorescence-3 DCM-PA** 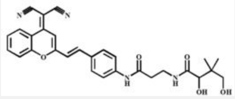	40 min			0.69 ng/mL	Cells and in vivo imaging	3.356 µM	195.2 nM/min /ng	(5)
**Electrochemical: VaninLP** 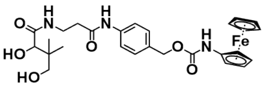	**30 min**	**Rapid, simple, sensitive, selective, in situ analysis in live cells**	**Also depends on 0.5% organic solvent**	**2.47 ng/mL**	**blood, urine, and FBS**	**2.38 µM**	**1.25 nM/min /ng**	**This work**

SI. 1. Materials and methods

All of the chemicals, including Calcium-D-pantothenate, trifluoroacetic acid, (TFA), (1S)-(+)-10-camphorsulfonic acid (CSA), anisaldehyde dimethyl acetal, N,N-diisopropylethylamine (DIPEA), anhydrous tetrahydrofuran, N-hydroxysuccinimide, 4-aminobenzyl alcohol, anhydrous N,N-dimethyl-formamide, anhydrous dichloromethane, anhydrous toluene, anhydrous ethanol, piperidine, N-ethyldiisopropylamine, were purchased from Sigma and TCI, recombinant human vanin-1/VNN1/pantetheinase is purchased from the U.S company R&D systems, a biotechne brand and the inhibitor RR6 were obtained from Sigma. The stock solution (1 μg/mL) of pantetheinase was prepared in ultrapure HEPES buffer and stored in small aliquots at −80 °C freezer to avoid repeated freeze−thaw cycles. The Institute of Biochemical and Biomedical Engineering at National Taipei University of Technology in Taiwan provided the HepG2 cells. A Bruker Avance III 300 or Bruker Avance III 500 spectrometer was used to get 1H NMR spectra at room temperature at the National Taipei University of Technology instrument centre or the Taipei Medical University instrument centre in Taiwan. The mass spectrometry performed by the facility of National Taiwan University; tiny compounds were subjected to electrospray ionisation (ESI) mass spectrometry using a Micromass LCT device. A CHI 611D electrochemical workstation operating in ambient conditions was used for all electrochemical investigations. The synthetic routes to the probe VaninLP (Scheme S1), and the spectral data collected to provide their chemical and structural characterizations are described in (Figures. S1-S8).

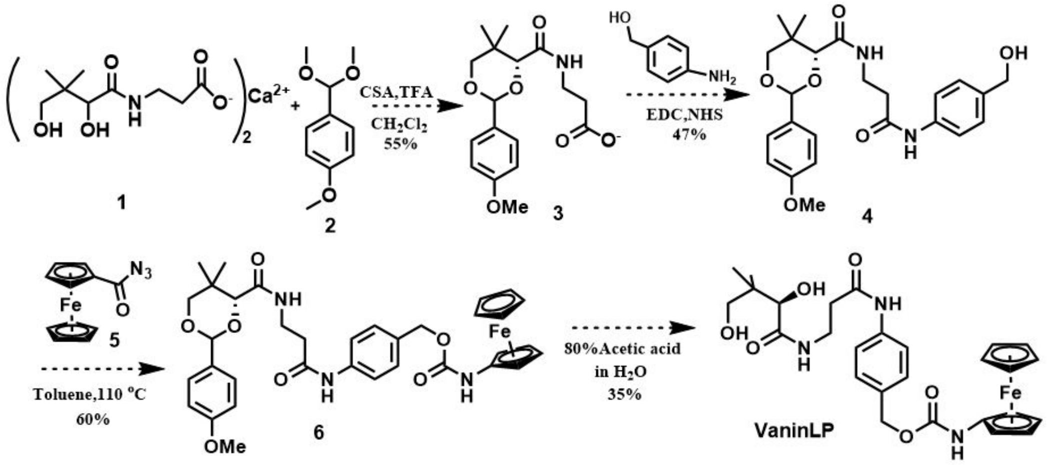

Scheme S1. Synthetic scheme of **VaninLP.**

Abbreviations:

CSA- Camphor sulfonic acid**,** **EDC**-1-ethyl-3-(3-dimethylaminopropyl)carbodiimide**,** **NHS**-N-hydroxy succiniimide**, DPPA-**diphenylphosphoryl azide**,** **DIPEA-** N,N-diisopropylethylamine**, DCM-**dichloromethane, **TFA-** Trifluoro acetic acid, **DMSO**-dimethyl sulfoxide, **EA**-ethyl acetate, **Hex-**hexanes, **EtOH**-ethanol, **$MgSO_4$**-magnesium sulfate.

SI. 1.1. Synthesis of compound 3. Compound **3** was prepared by following the procedure reported by Yang et al. 2020.[6] In an oven-dried 2-neck round-bottomed flask, 93.5 mg of CSA, 950 mg of compound **1** were added, and then 15 ml of TFA was added dropwise while stirring at 0^oC. After all solids were dissolved, the trifluoroacetic acid was evaporated under reduced pressure to give a colorless oil. Next, 2.25 g of 4-methoxybenzaldehyde dimethyl vinyl ether was added to the oil containing 20 ml of dichloromethane (DCM), and stirred at room temperature for 8 hours. After the reaction, 30 ml of 1 M sodium carbonate was added and the precipitate was filtered. The filtrate was then acidified to pH 4.5 with acetic acid, and the product in the solution was extracted with dichloromethane. After the separated dichloromethane was dried with anhydrous magnesium sulfate (MgSO4), the solvent was removed by evaporation to obtain compound (2) as a white solid (1.3 g, yield 55%), which was used in the following step without further purification step. ^1H NMR(300MHz,$CDCl_3$) δ 7.49 – 7.21 (m, 2H), 6.98 (d, J = 36.1 Hz, 1H), 6.89 (d, J = 8.8 Hz, 2H), 5.44 (s, 1H), 4.08 (s, 1H), 3.78 (s, 3H), 3.66 (q, J = 11.5 Hz, 2H), 3.51 (dd, J = 12.8, 6.3 Hz, 2H), 2.58 (t, J = 6.1 Hz, 2H), 0.97 (d, J = 56.1 Hz, 6H) (Figure S1).

SI. 1.2. Synthesis of compound 4. Compound **4** was prepared by following the procedure reported by our group (7). Take compound (2) (1.00 g, 0.244mmol, 1 eq), N-Hydroxysuccinimide (NHS) (0.42 g, 0.365mmol, 1.5 eq) and 1-(3-Dimethylaminopropyl)-3-ethylcarbodiimide hydrochloride (EDC) (0.7 g, 0.365 mmol, 1.5 eq) in a double-necked flask, add anhydrous dichloromethane (DCM) (10 mL), stirred at room temperature overnight under nitrogen, Extract using Ethyl acetate (EA) (10 mL), dilute with water (2×20 mL), collect the organic layer and dried using magnesium sulfate. And remove the organic solvent under pressure to obtain a white solid crude product. The crude product does not need to be purified. It is directly placed in a double-necked flask with 4-Aminobenzyl alcohol (0.533 g, 0.488 mmol, 2eq), anhydrous Dichloromethane (DCM) (20 mL) is added, stirred at room temperature overnight under nitrogen, and then extracted with DCM and water for 3 times, collect the organic layer and dried using magnesium sulfate. After filtering out the magnesium sulfate, remove the organic solvent by reduced pressure. The crude product was purified by flash chromatography on silica gel (Rf = 0.4, Eluent: EA/Heaxane = 1/1, v/v), the light yellow foamy product compound (4) (0.855 g) can be obtained, with a yield of 47%.^1H

NMR(300MHz,$CDCl_3$) δ 8.025 (s, 1H), 7.53 (d, J = 8.6 Hz, 2H), 7.50 – 7.24 (m, 4H), 7.13 – 7.07 (m, 1H), 6.87 (d, J = 8.7 Hz, 2H), 5.43 (s, 1H), 4.63 (s, 2H), 4.08 (s, 1H), 3.8 (s, 3H), 3.69 (d, J = 6.4 Hz, 4H), 2.64 (s, 2H), 1.10 (s, 6H) (Figure S2).

SI. 1.3. Synthesis of compound 6. Compound **6** was prepared according to earlier reports.[7] Dissolve compound (**4**) (0.80 g, 0.155 mmol, 1eq) and compound (**5**) (0.55 g, 0.187 mmol, 1.2 eq) in Toluene (4 mL), place it in a double-necked flask, and set up a reflux tube under nitrogen conditions and stirred at 90°C for 2.5 hours. After the reaction, add silica powder and use a rotavaporator to remove the organic solvent. Finally, use column chromatography to purify (Rf = 0.6, eluent: EA/Hexane= 3/4, v/v) The brownish red-colored solid compound (**6**) (0.588 g) can be obtained with a yield of 60%. ^{1}H NMR (300 MHz, $CDCl_3$) δ 8.46 (s, 1H), 7.51 (d, *J* = 7.8 Hz, 2H), 7.37 (d, *J* = 8.2 Hz, 2H), 7.33 – 7.23 (m, 2H), 7.11 (s, 1H), 6.86 (d, *J* = 8.3 Hz, 2H), 6.24 (s, 1H), 5.41 (s, 1H), 5.27 (s, 1H), 5.07 (s, 2H), 4.45 (s, 2H), 4.08 (d, *J* = 15.9 Hz, 6H), 3.94 (s, 2H), 3.79 (s, 3H), 3.63 (d, *J* = 7.4 Hz, 2H), 3.59 – 3.48 (m, 2H), 2.56 (s, 2H), 1.05 (s, 6H) (Figure S3). Calculated for chemical formula: $C_{35}H_{39}FeN_3O_7$: m/z 669.21, found 669.21(M^+), 692.20 ($M+Na^+$), 708.18 ($M+K^+$) (Figure S4). IR spectra: 3400, 3004, 1695, 1545, 1210, 1000-650 (Figure S5).

SI. 1.4. Synthesis of compound VaninLP. Compound **VaninLP** was prepared by dissolve compound (6) (130mg, 0.25 mmol, 1 eq) in 80% acetic acid in H_2O (1 mL) in a single-neck round-bottomed flask, stir at room temperature under nitrogen for 2 hours, then extract and Wash with K_2CO_3 and remove the organic solvent with reduced pressure, and then purify with column chromatography (Rf = 0.2, eluent: MeOH/DCM = 1/4, v/v) to obtain the yellow crystal product **VaninLP** (118 mg) with 35% of yield. ^{1}H NMR (500 MHz, MeOD) δ 7.56 (d, *J* = 8.2 Hz, 2H), 7.34 (d, *J* = 7.8 Hz, 2H), 5.08 (s, 2H), 4.52 (s, 2H), 4.06 (d, *J* = 70.3 Hz, 6H), 3.88 (s, 2H), 3.71 – 3.46 (m, 2H), 3.44 (d, *J* = 10.9 Hz, 1H), 3.37 (d, *J* = 10.9 Hz, 1H), 3.30 (dt, *J* = 3.2, 1.6 Hz, 4H), 2.61 (t, *J* = 6.5 Hz, 2H), 0.89 (d, *J* = 1.0 Hz, 6H) (Figure S6). Calculated for chemical formula: $C_{27}H_{33}FeN_3O_6$: m/z 551.17, found 551.17(M^+), 574.16 ($M+Na^+$), 590.13 ($M+K^+$) (Figure S7). IR spectra: 3450, 3004, 1695, 1545, 1210, 1000-650 (Figure S8).

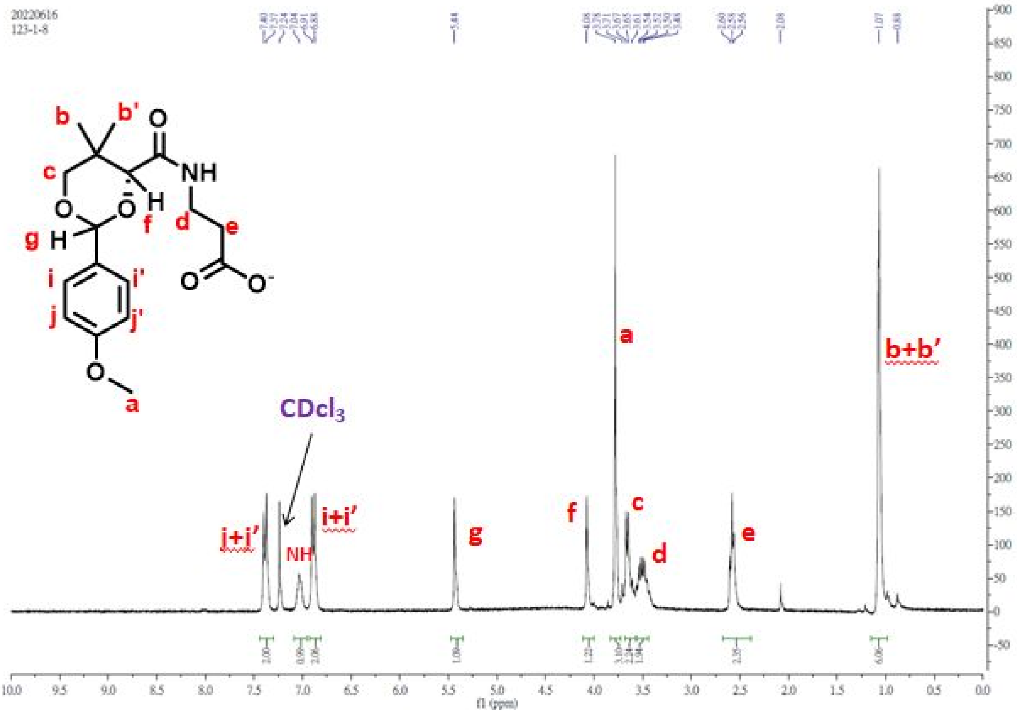

Figure S1. ^{1}H NMR spectrum of compound-3.

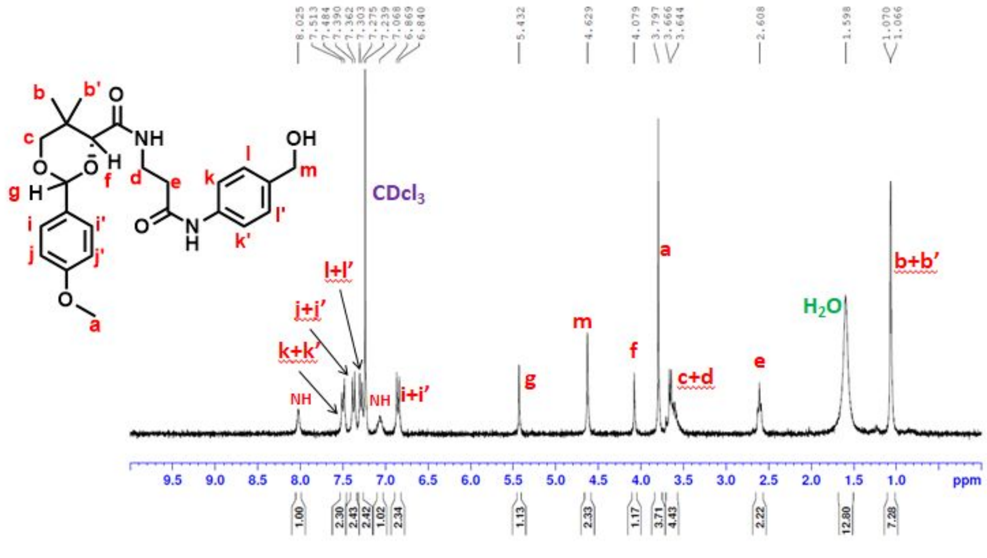

Figure S2. ^{1}H NMR spectrum of compound-4.

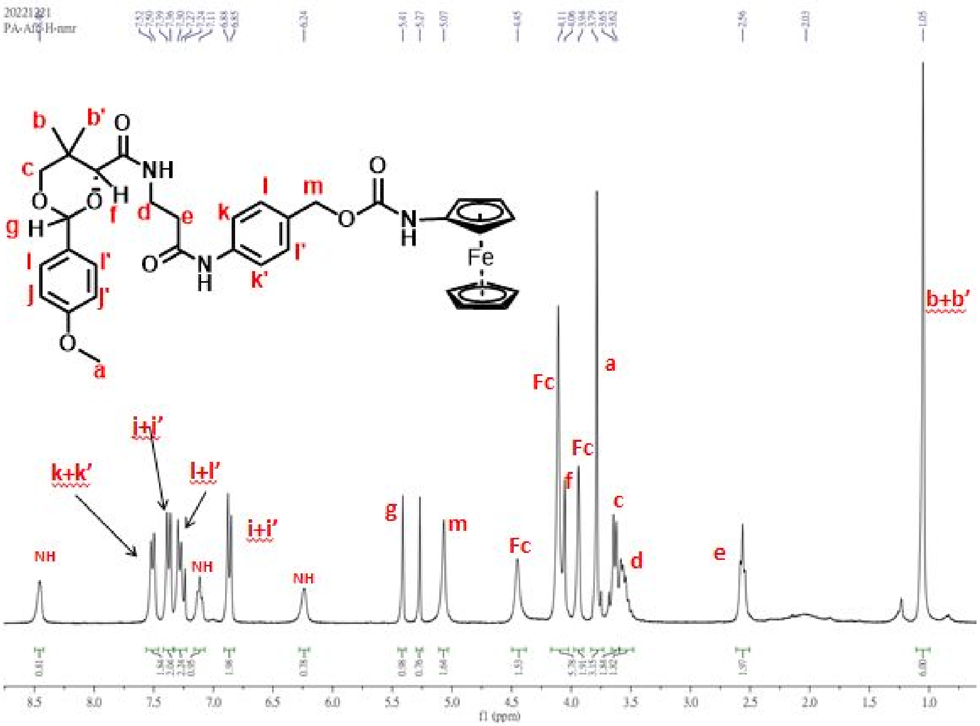

Figure S3. ^{1}H NMR spectrum of compound-6.

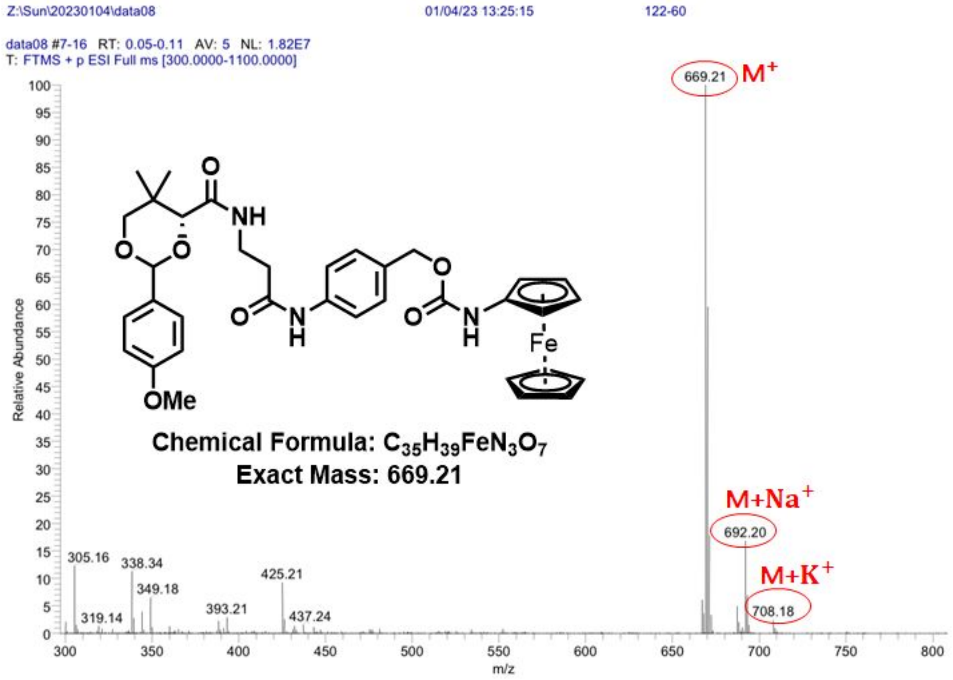

Figure S4. Mass spectrum of compound-6.

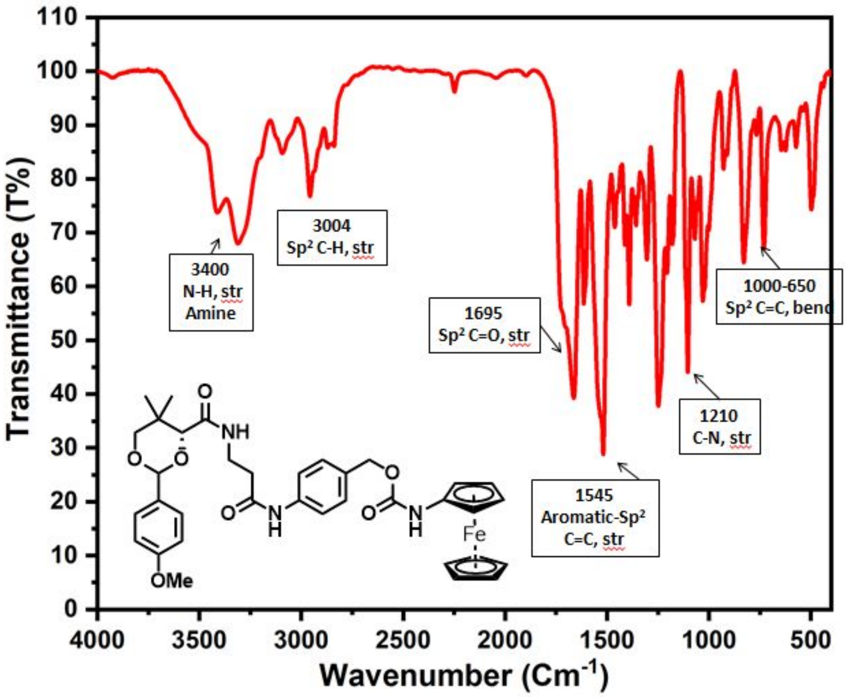

Figure S5. FT-IR spectrum of compound-6

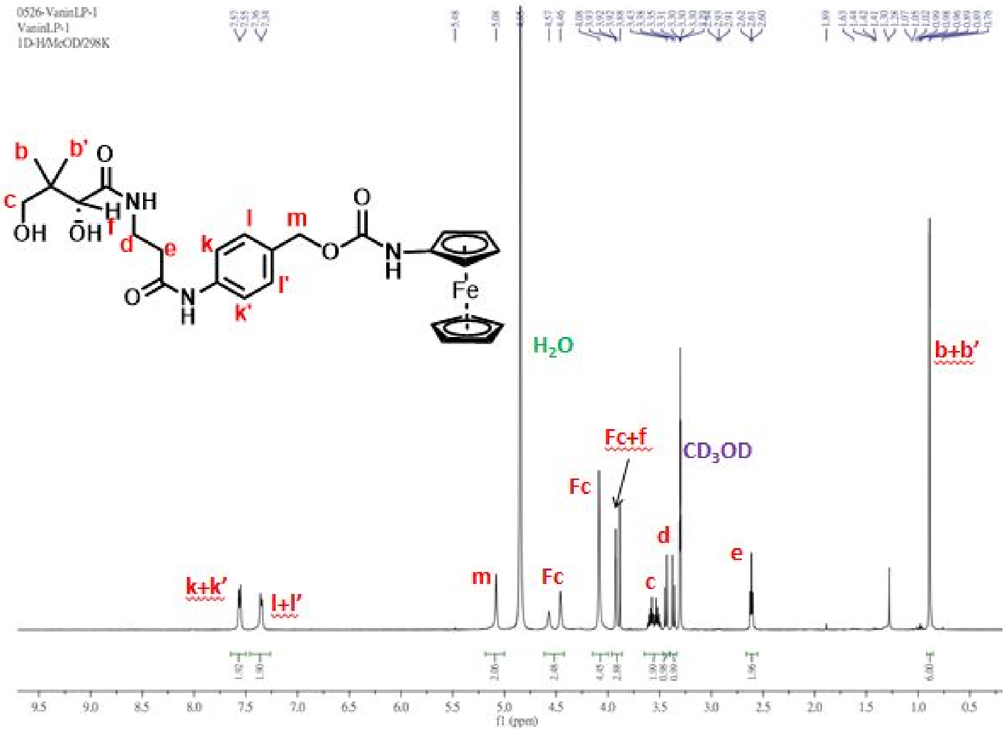

Figure S6. ^{1}H NMR spectrum of compound-VaninLP

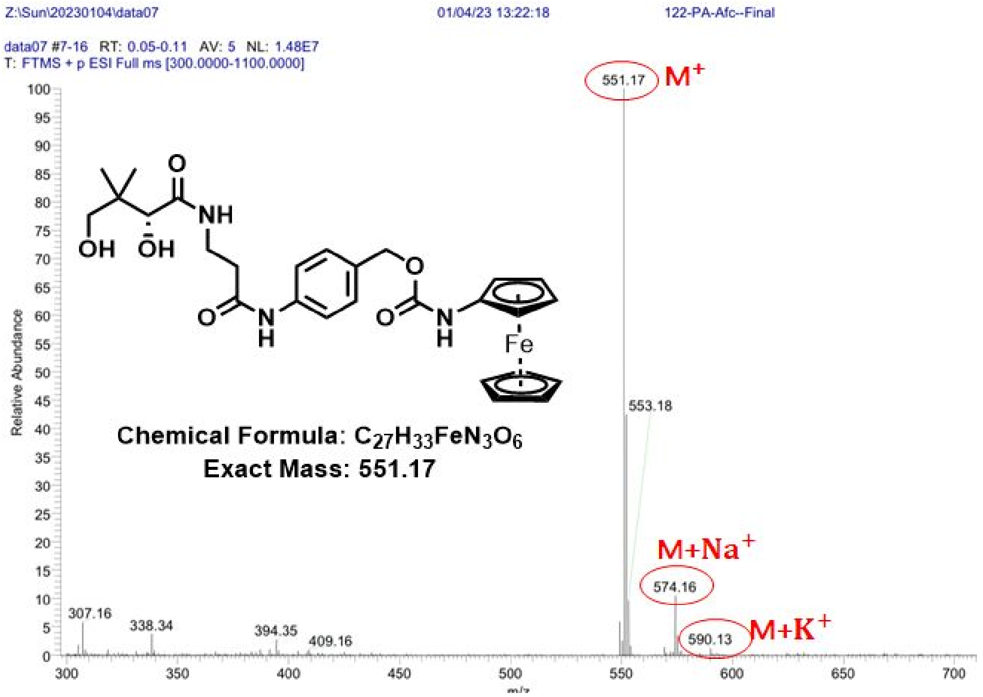

Figure S7. Mass spectrum of compound-VaninLP

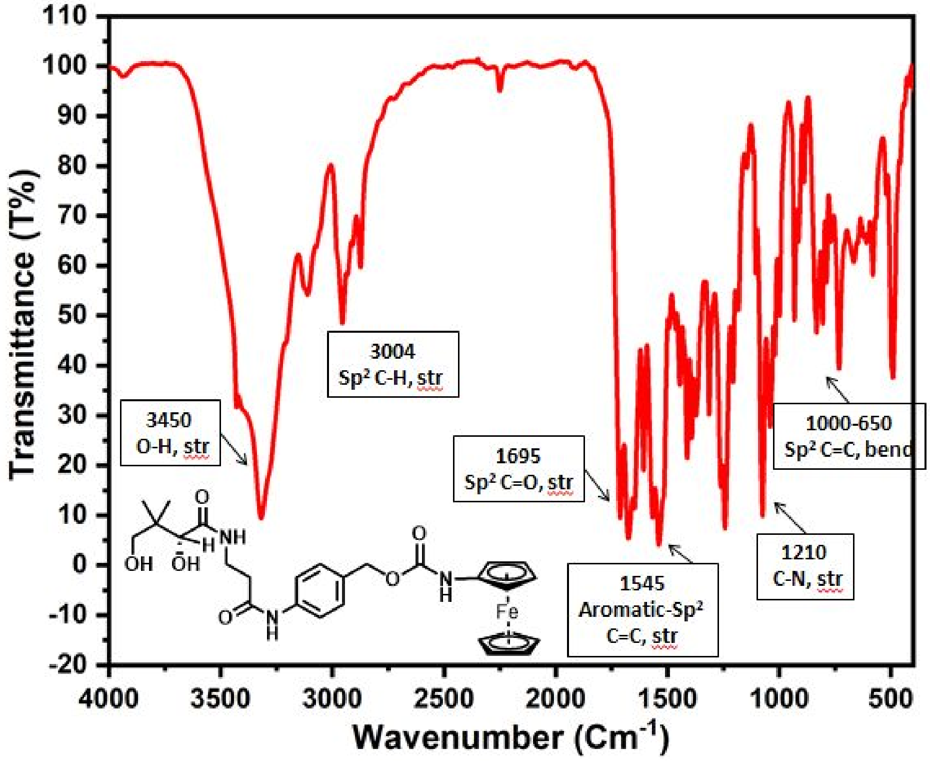

Figure S8. FT-IR spectrum of compound-VaninLP

SI. 2. Procedure for cell culture and MTT assay

Human liver cancer HepG2 cells were grown in a culture dish in modified Eagle medium (MEM) containing 10% fetal bovine serum (FBS) and 1% antibiotics (penicillin, streptomycin, amphotericin). In a humidified environment containing 5% CO_2 at 37°C, the cells were cultured until they occupied 90% of the culture dish, then the culture medium was removed, 10 mL of PBS buffer was added and removed again. Add 1 mL Trypsin and place it back in the 37°C incubator to react for 5 minutes. Tap the culture dish gently to suspend the cells, then add 1 mL of culture medium to neutralize the Trypsin. Take the mixed solution into a 15 mL centrifuge tube and incubate at 1000 rpm at 4°C. Centrifuge for 5 minutes, then remove the supernatant and add 1 mL of culture medium to redissolve. Take out 10 μL of cell solution and mix with 10 μL of Trypan blue for counting. Take out the required amount of cell solution ($1\times10^3 \sim 1\times10^6$), and fill the volume with culture medium to 1 mL. Add cells at 1×10^6 cells/mL to a 24-well plate and culture at 37°C overnight to allow the cells to adhere to the plate and then remove the culture medium. The blank control group added 1 μL DMSO and 990 μL Hepes buffer (0.1 M, pH7.0); the experimental group VaninLP: added 50 μM VaninLP (0.5 μL of VaninLP (stock:20 mM in DMSO)), 999 μL Hepes buffer (0.1 M, pH7.0). And then remove all solutions after incubation at 37°C for 2, 4, and 6 hours respectively. Add MTT (5 mg/mL in PBS) and react in the dark at 37°C for 3 hours before removing. Add quantitative IPA to dissolve the purple crystals and then use an ELISA reader to measure the absorbance at 562 nm and recorded. Then compared with just the cultue medium alone.

$$\text{Cytotoxicity (\%)} = \frac{\text{OD}_{\text{GTLP /GTLPOH}}}{\text{OD}_{\text{blank}}} \times 100\%$$

SI. 3. Preparation of electrode and Voltammetric parameters

In electrochemical sensing experiments, a three-electrode system was used: a platinum wire was used as the counter electrode, a glassy carbon electrode (GCE) served as the working electrode (electroactive surface area = 0.071 cm^2), and saturated Ag/AgCl (saturated KCl) served as the reference electrode. Electrochemical tests were carried out under ambient conditions (pH 7; 37°C) using DMSO/HEPES (1% (volume/volume)). At a scan rate of 0.05 V/s, cyclic voltammetric tests were conducted in the potential range of -0.3 to +0.6 V. In the potential window of -0.3 to +0.4 V, differential pulse voltammetry was used with an amplitude of 0.05 V, pulse width of 0.05 s, and quiet time of 2 s. Every experiment was run in triplicate, and the figures were plotted using the average values.

SI. 4. Cleaning procedure of working electrode

The working electrode (GCE) was cleaned using three different alumina neutral slurry messes (0.5, 0.3, and 0.05 microns). After the cleaning process was finished, the electrode was repeatedly washed with an excessive amount of DI water in order to remove the adsorbed alumina particles on the surface of GCE, after getting clear control experiments; electrode can be used for electrochemical characterisation and analysis.

SI. 5. Preparation of stock solution

The ideal concentrations of HEPES powder and NaOH pellets were used to make 0.1M HEPES buffer solution, pH 7. A stock solution of 10 mM VaninLP was prepared in DMSO. A stock solution about 1μg/mL, 0.1 μg/mL and 0.01μg/mL of pantetheinase enzyme was produced in 0.1 M HEPES buffer solution. Using a 0.1 M HEPES solution, 50% of the blood and 20% of urine samples (v/v) were diluted.

SI. 6. Optimizing analytical condition

The pH and the percentage of DMSO of the system has a significant impact on the electrochemical response and the viability of chemical and biological reactions. Effective sensing platforms require pH values and the percentage of DMSO to be optimized in order to maximize electrochemical signals and reaction speeds. This were demonstrated by examining the electroanalytical performance of the ratiometric probe VaninLP, in various pH-varying electrolytes while 25ng/mL of pantetheinase and 0.5 % DMSO (volume/volume) were present (Figures. S9A&B). When buffer systems and DMSO percentage were evaluated, the response current at pH 7 at 0.1 M HEPES buffer solution and 0.5 % DMSO was the highest. Accordingly, the perfect supporting electrolyte for pantetheinase electrochemical sensing is 0.1 M HEPES buffer solution.

Since increasing the volume of blood virtually had no effect on the outcome electrochemical response, the fraction of blood samples was tuned to be 50% (volume/volume). The presence of excess non-ionizable constituents in blood may have caused the concentration of the supporting electrolyte to change, leading to a decrease in electrical conductivity. This could explain why the best electrochemical signal responses were observed when 50% (volume/volume) blood was added to the DMSO/HEPES buffer solution mixture (1% v/v), values commensurate with those of a standard HEPES buffer. The 1mL aliquots containing 50 μM VaninLP probe towards 25 ng mL^{-1} of pantetheinase in DMSO/HEPES (0.5/99.5%, pH 7) were incubated over 120 min under ambient conditions and analyzed via DPV (Figure S9C). As result, shows an increase in the current response

of AF from 5 min to 30 min, after 30 min the current response gradually reduced. Moreover, a significant response current was obtained at 30 min, so we operate the samples were incubated for 30 min for further experiments.

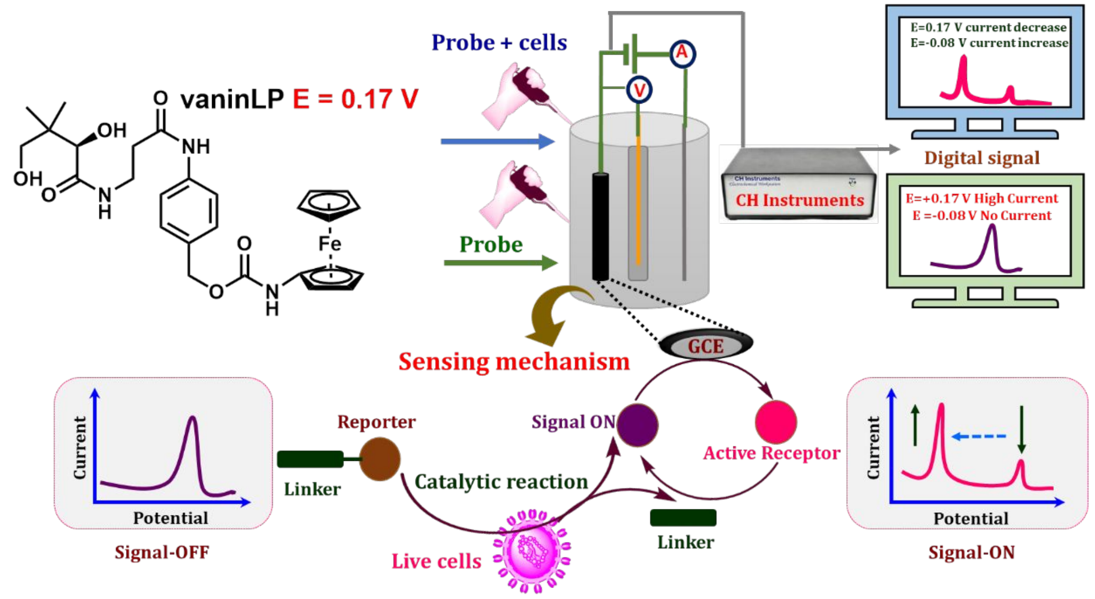

Scheme S2. A schematic representation of electrochemical sensing of pentatheinase activity on the surface of an electrode.

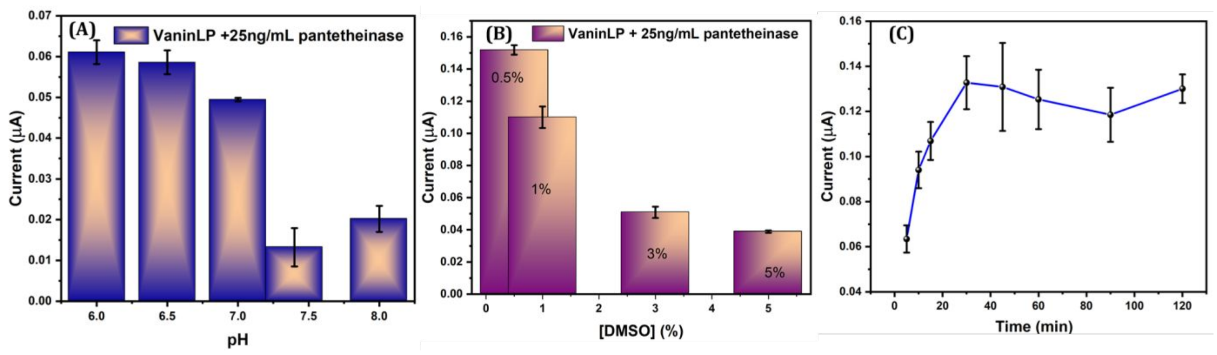

Figure S9. (A) Bar chart of current responses of the VaninLP probe for different pH values. (B) Bar chart of the VaninLP probe's current responses for different percentages of DMSO. (C) A Plot of current response of the VaninLP probe for different incubation time. All samples were in DMSO/HEPES (volume/volume).

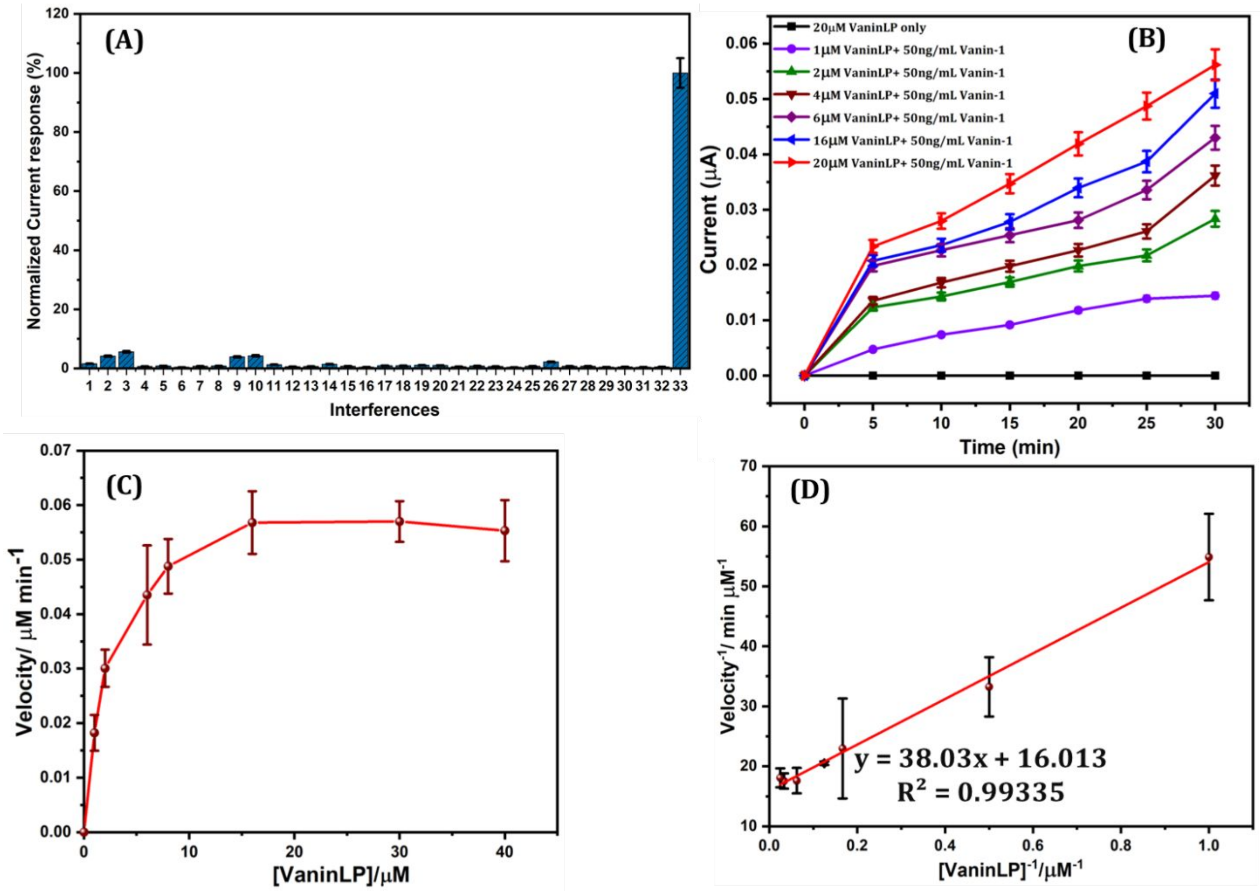

Figure S10. (A) Electrochemical responses of VaninLp (50 μM) probe with different species 1 to 33 (From right to left) 1. Ascorbic acid, 2. Dopamine, 3. Uric acid, 4. L-cysteine, 5. Glutathione, 6. Glucose, 7. Creatine, 8. Urea, 9. Copper chloride, 10. Valine, 11. Phenylalanine, 12. Tryptophan, 13. Asparagine, 14. Threonine, 15. Sarcosine, 16. Calcium pantathienate, 17. Alanine, 18. Magnesium chloride, 19. Lysine, 20. Ferrous chloride, 21. Glycine, 22. Proline, 23. Arginine, 25. Calcium chloride, 26. GGT, 27. Esterase, 28. Leucine aminopeptidase, 29. H_2O_2, 30. DPP-IV, 31. Trypsin, 32. APN 33. Pantetheinase respectively. **(B)** The kinetic plot of current response versus reaction time curves acquired using VaninLP probe at various concentration (1-20 μM/mL) in the absence and presence of pantetheinase. **(C)** Michaelis-Menten plot of the reaction rate versus VaninLP concentration **(D)** Lineweaver-Burk plot of reciprocal of the reaction rate versus VaninLP concentrations.

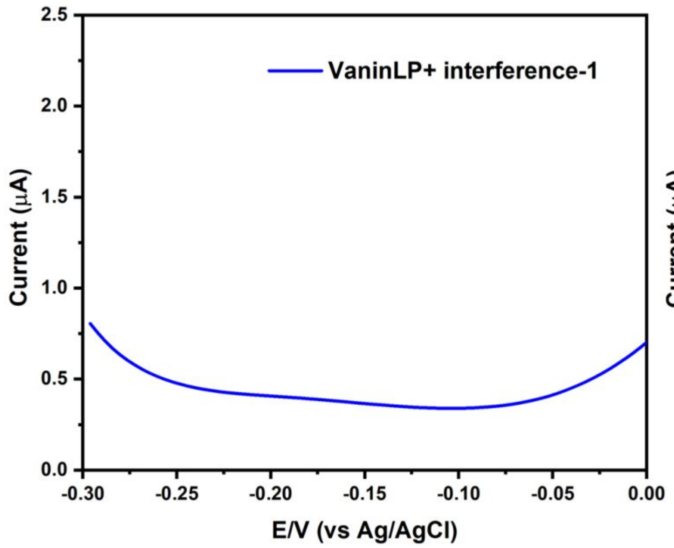
VaninLP+ interference-1
Current (μA)
2.5
2.0
1.5
1.0
0.5
0.0
-0.30
-0.25
-0.20
-0.15
-0.10
-0.05
0.00
E/V (vs Ag/AgCl)

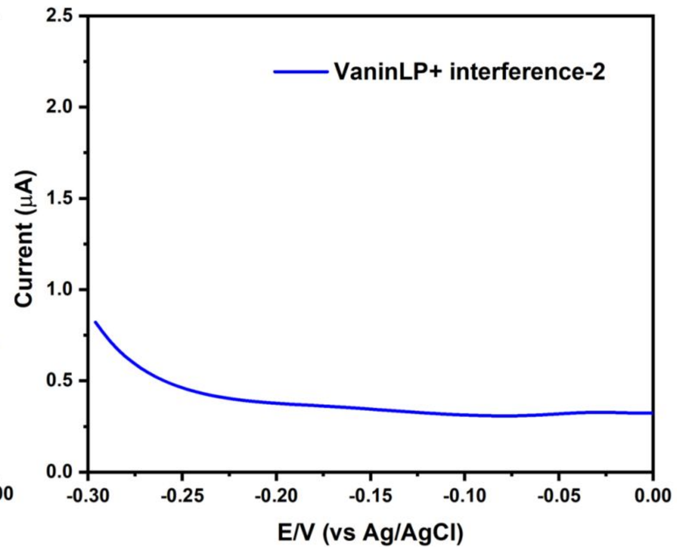
VaninLP+ interference-2
Current (μA)
2.5
2.0
1.5
1.0
0.5
0.0
-0.30
-0.25
-0.20
-0.15
-0.10
-0.05
0.00
E/V (vs Ag/AgCl)

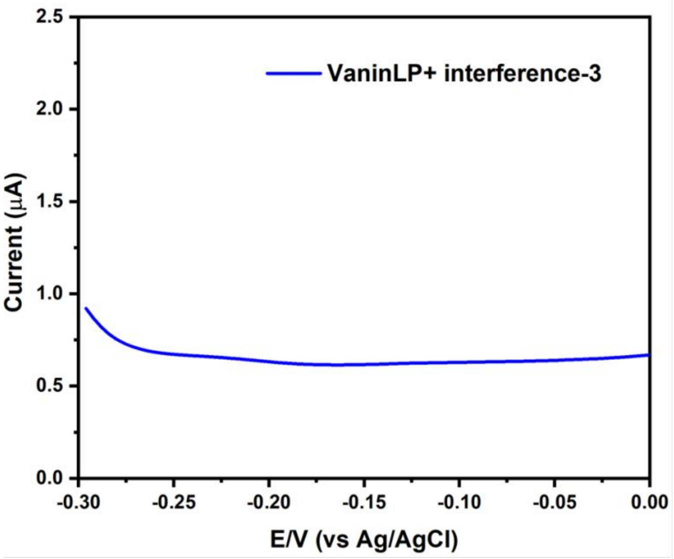
VaninLP+ interference-3
Current (μA)
2.5
2.0
1.5
1.0
0.5
0.0
-0.30
-0.25
-0.20
-0.15
-0.10
-0.05
0.00
E/V (vs Ag/AgCl)

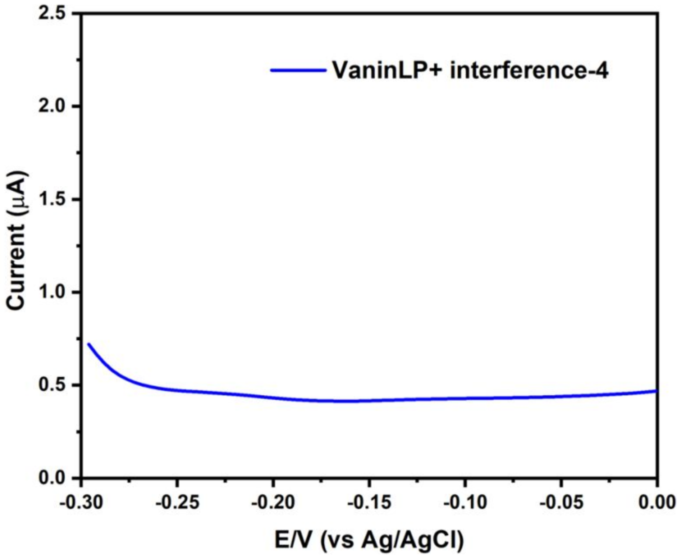
VaninLP+ interference-4
Current (μA)
2.5
2.0
1.5
1.0
0.5
0.0
-0.30
-0.25
-0.20
-0.15
-0.10
-0.05
0.00
E/V (vs Ag/AgCl)

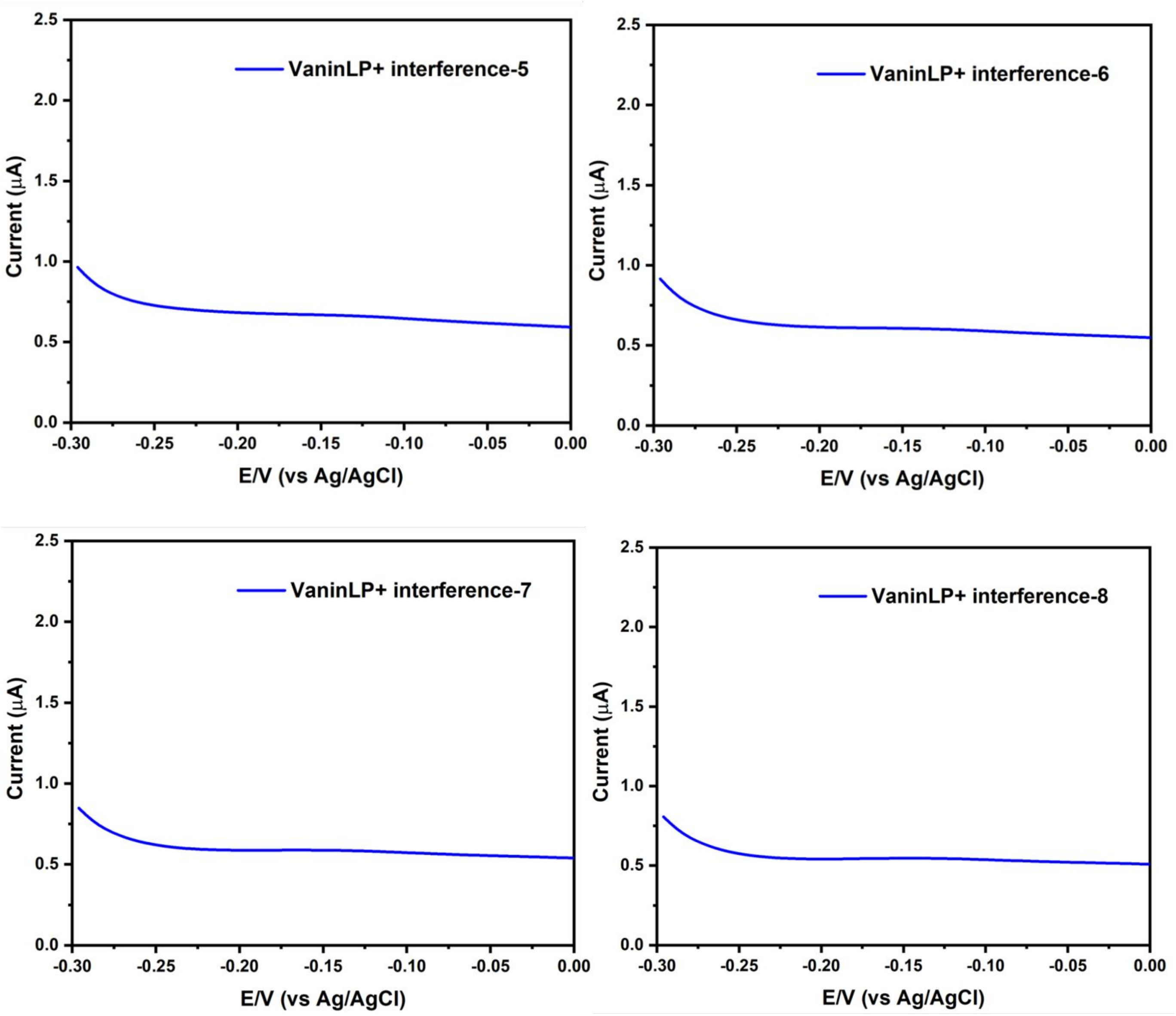
VaninLP+ interference-5
VaninLP+ interference-6
VaninLP+ interference-7
VaninLP+ interference-8
Current (μA)
E/V (vs Ag/AgCl)
0.0
0.5
1.0
1.5
2.0
2.5
-0.30
-0.25
-0.20
-0.15
-0.10
-0.05
0.00

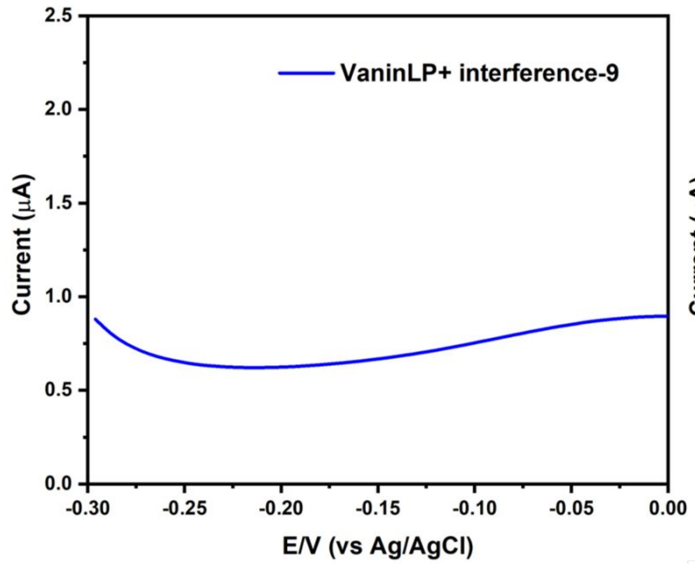
VaninLP+ interference-9
Current (μA)
E/V (vs Ag/AgCl)

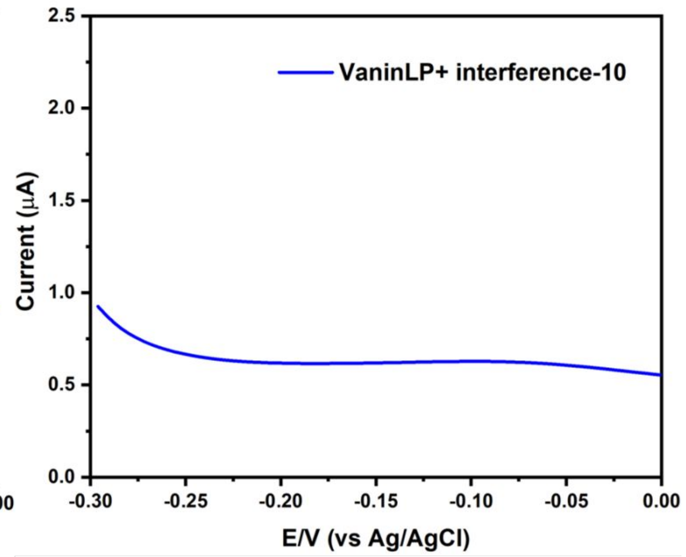
VaninLP+ interference-10
Current (μA)
E/V (vs Ag/AgCl)

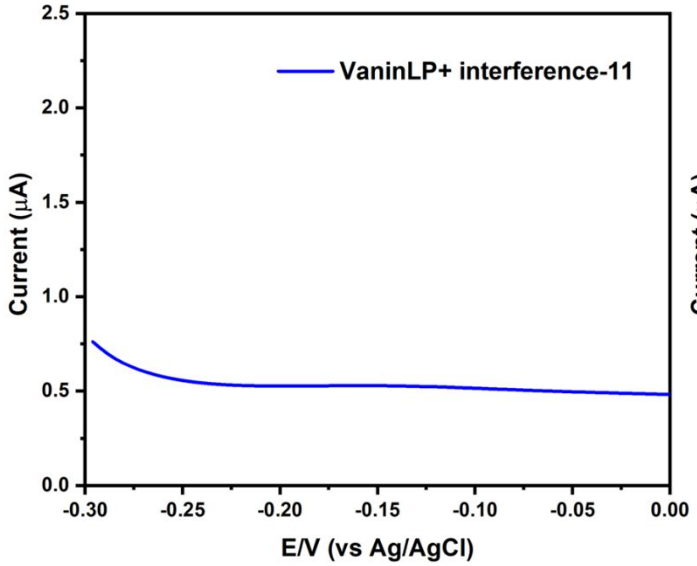
VaninLP+ interference-11
Current (μA)
E/V (vs Ag/AgCl)

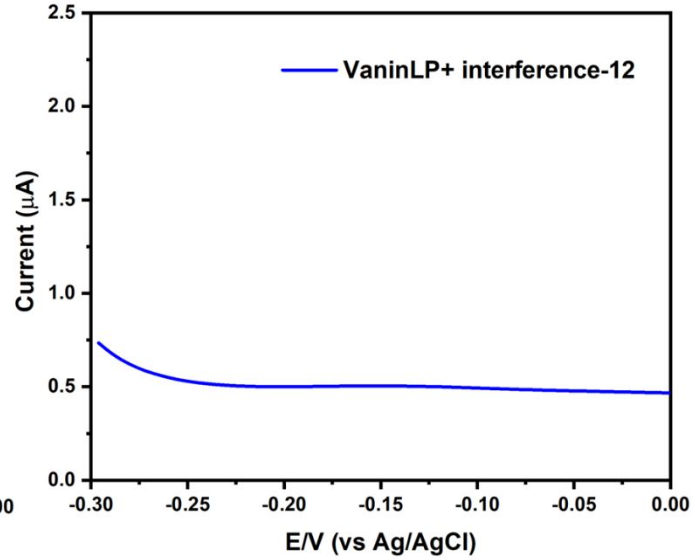
VaninLP+ interference-12
Current (μA)
E/V (vs Ag/AgCl)

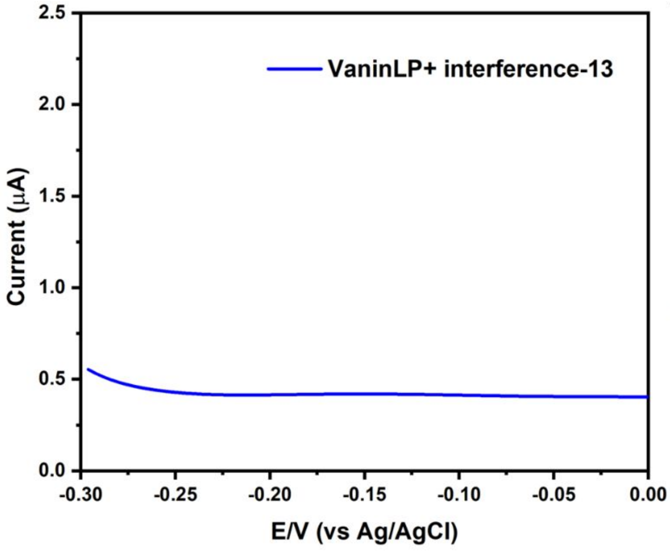
VaninLP+ interference-13
Current (μA)
2.5
2.0
1.5
1.0
0.5
0.0
-0.30
-0.25
-0.20
-0.15
-0.10
-0.05
0.00
E/V (vs Ag/AgCl)

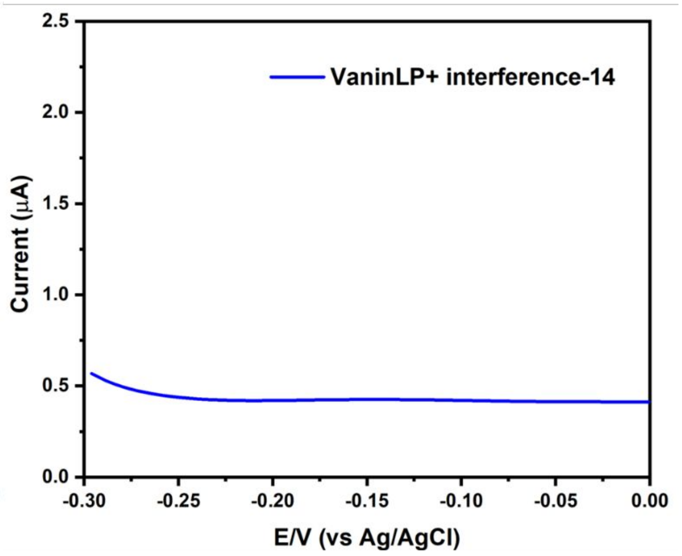
VaninLP+ interference-14
Current (μA)
2.5
2.0
1.5
1.0
0.5
0.0
-0.30
-0.25
-0.20
-0.15
-0.10
-0.05
0.00
E/V (vs Ag/AgCl)

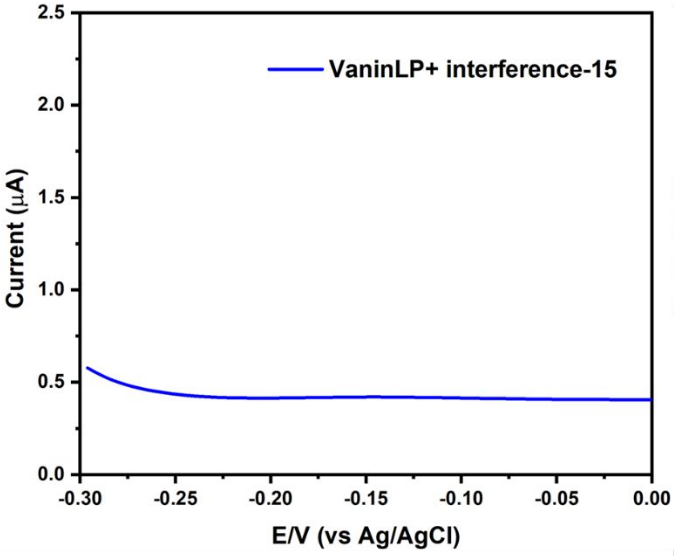
VaninLP+ interference-15
Current (μA)
2.5
2.0
1.5
1.0
0.5
0.0
-0.30
-0.25
-0.20
-0.15
-0.10
-0.05
0.00
E/V (vs Ag/AgCl)

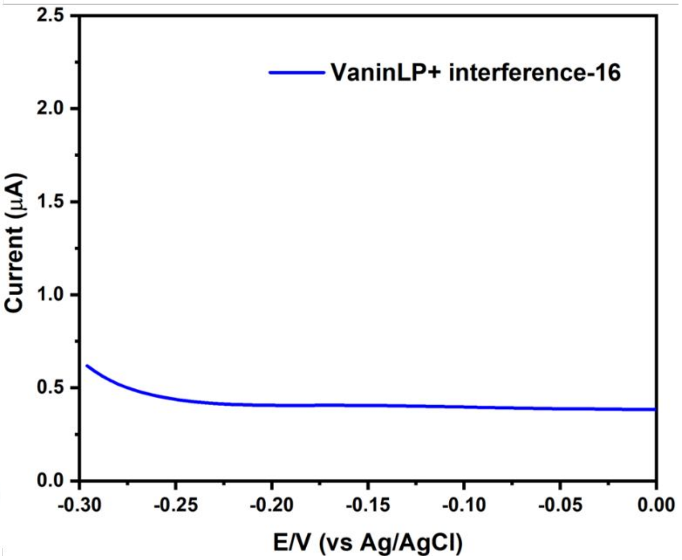
VaninLP+ interference-16
Current (μA)
2.5
2.0
1.5
1.0
0.5
0.0
-0.30
-0.25
-0.20
-0.15
-0.10
-0.05
0.00
E/V (vs Ag/AgCl)

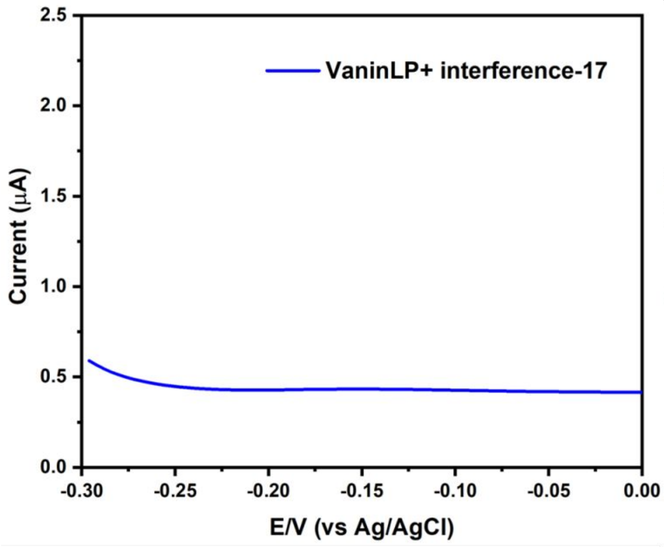
VaninLP+ interference-17
Current (μA)
E/V (vs Ag/AgCl)

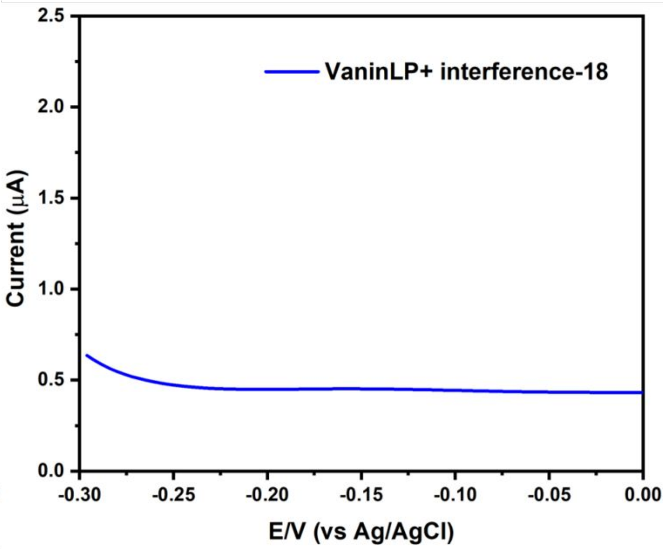
VaninLP+ interference-18
Current (μA)
E/V (vs Ag/AgCl)

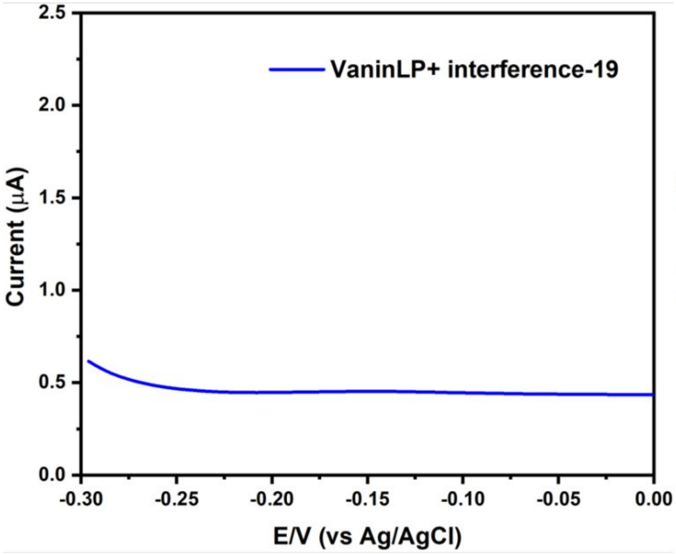
VaninLP+ interference-19
Current (μA)
E/V (vs Ag/AgCl)

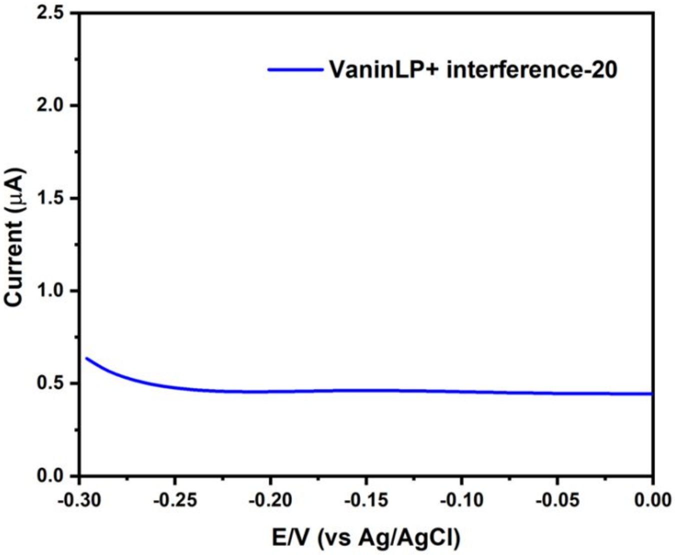
VaninLP+ interference-20
Current (μA)
E/V (vs Ag/AgCl)

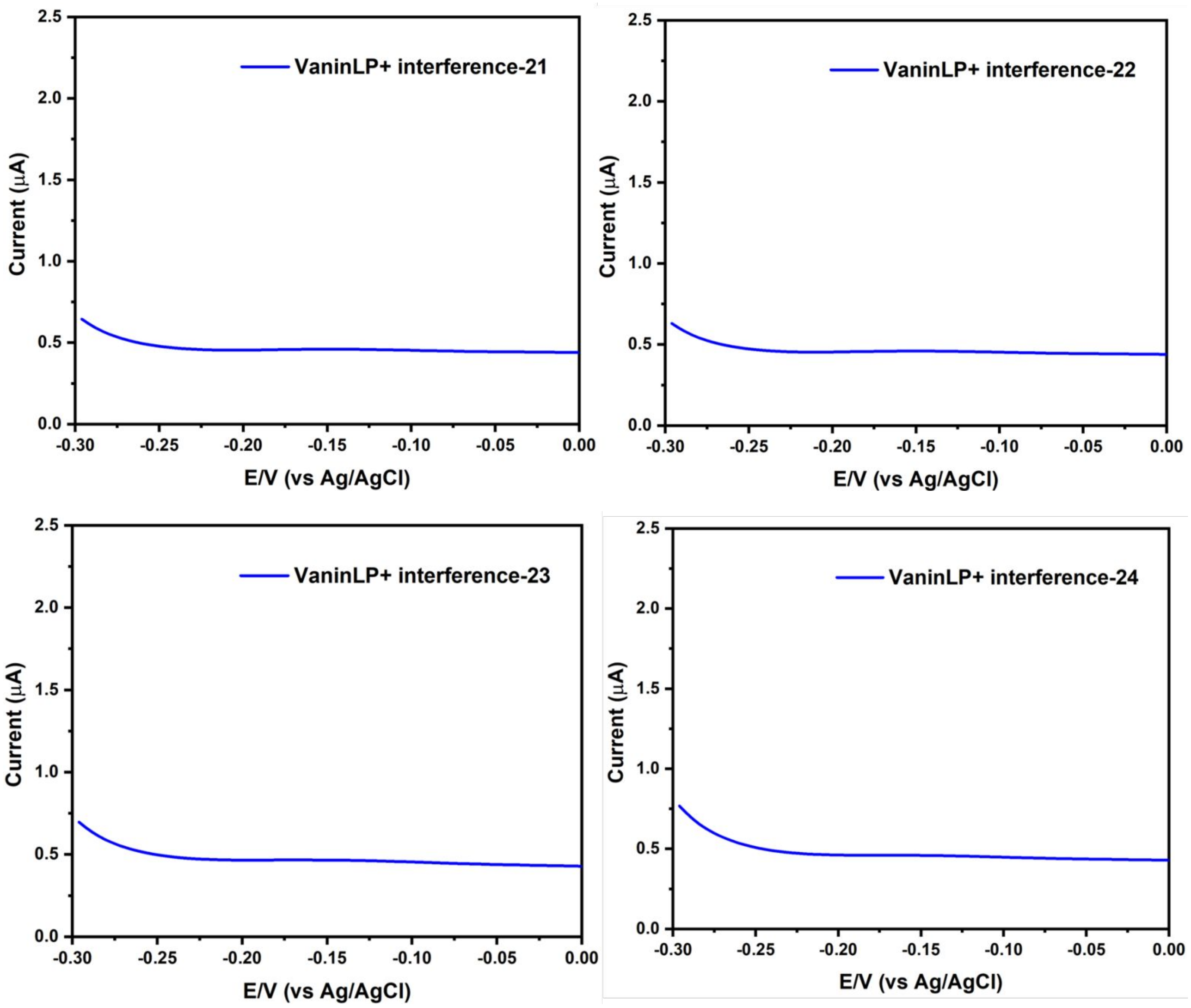
VaninLP+ interference-21
VaninLP+ interference-22
VaninLP+ interference-23
VaninLP+ interference-24
Current (μA)
E/V (vs Ag/AgCl)
0.0
0.5
1.0
1.5
2.0
2.5
-0.30
-0.25
-0.20
-0.15
-0.10
-0.05
0.00

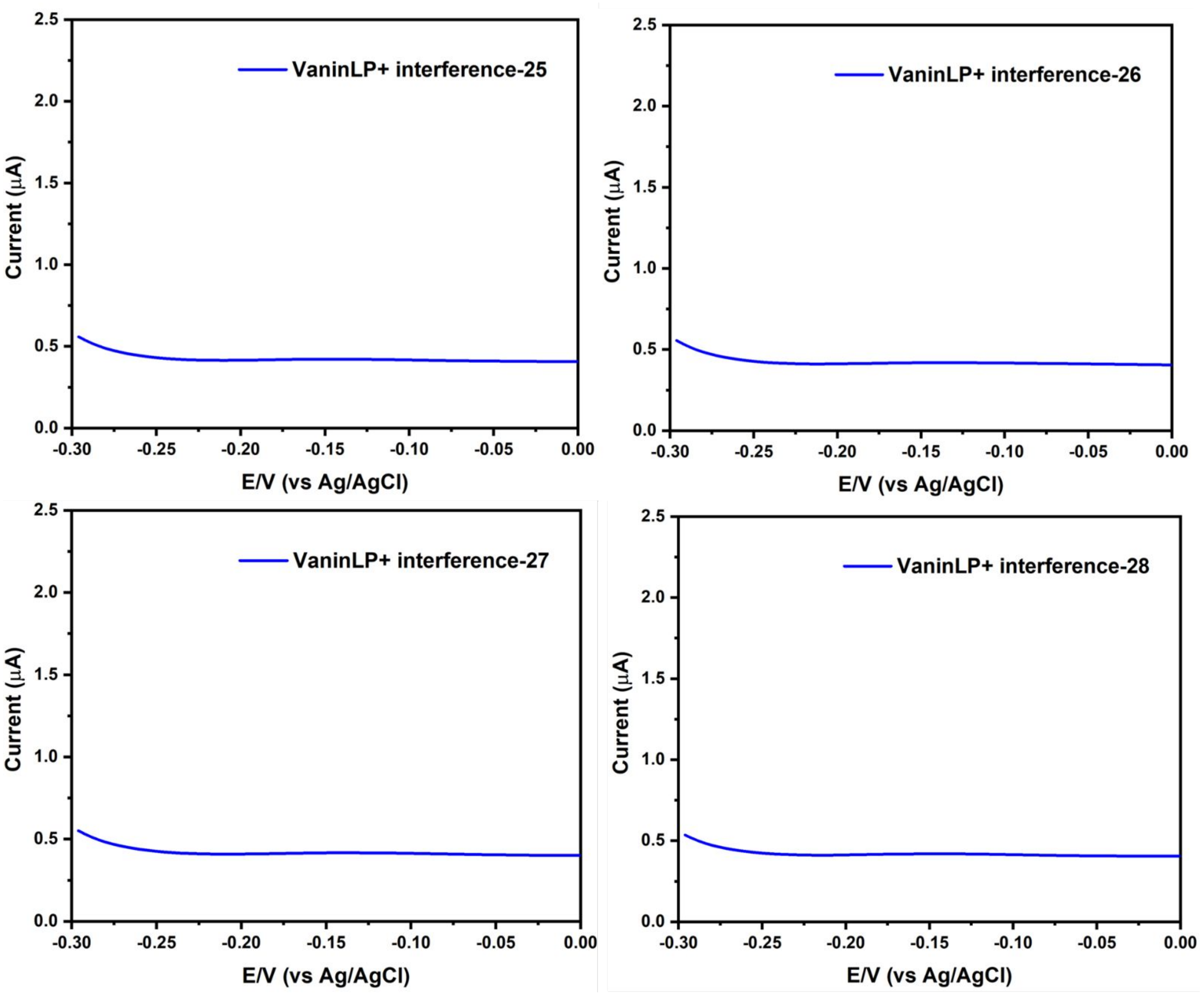
VaninLP+ interference-25
VaninLP+ interference-26
VaninLP+ interference-27
VaninLP+ interference-28
Current (μA)
E/V (vs Ag/AgCl)
2.5
2.0
1.5
1.0
0.5
0.0
-0.30
-0.25
-0.20
-0.15
-0.10
-0.05
0.00

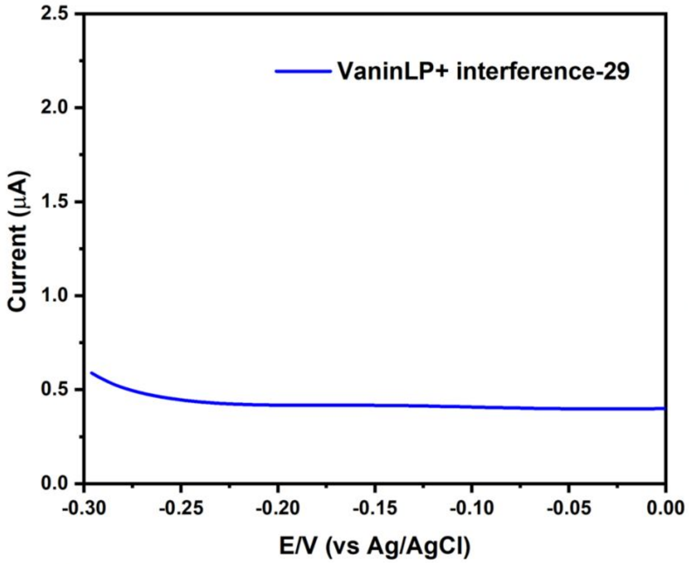
VaninLP+ interference-29
Current (μA)
E/V (vs Ag/AgCl)

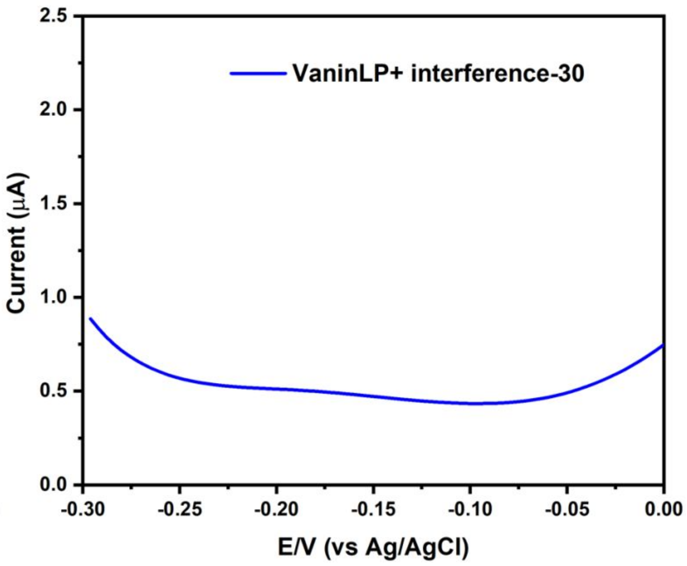
VaninLP+ interference-30
Current (μA)
E/V (vs Ag/AgCl)

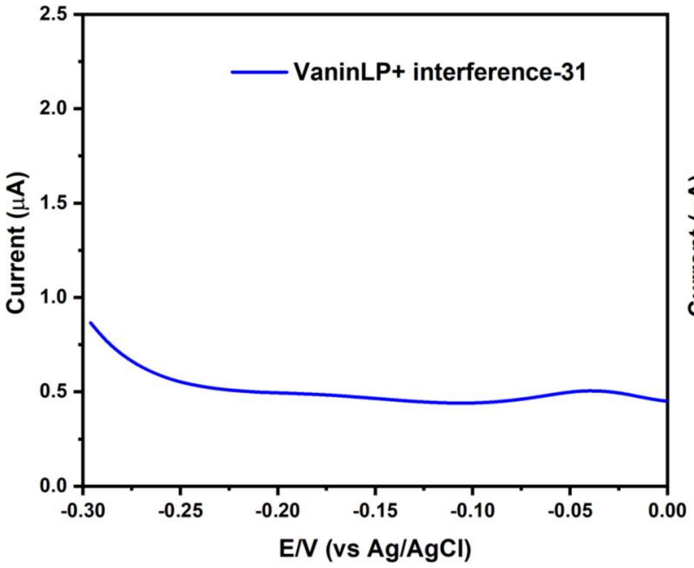
VaninLP+ interference-31
Current (μA)
E/V (vs Ag/AgCl)

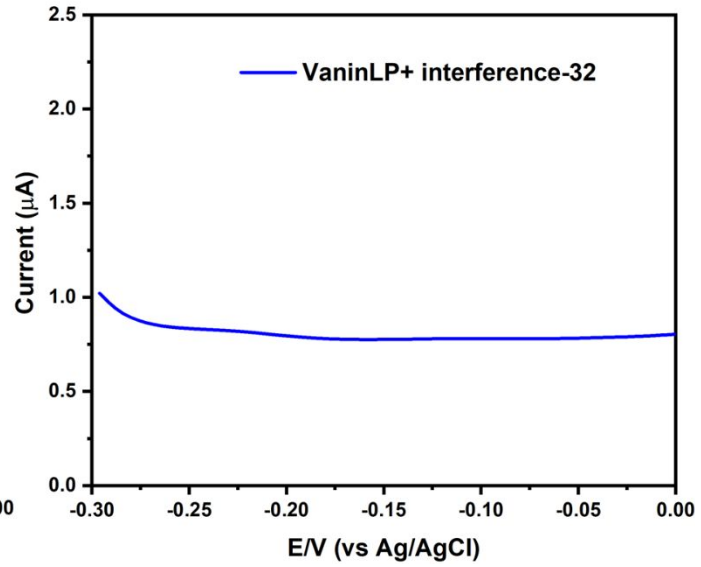
VaninLP+ interference-32
Current (μA)
E/V (vs Ag/AgCl)

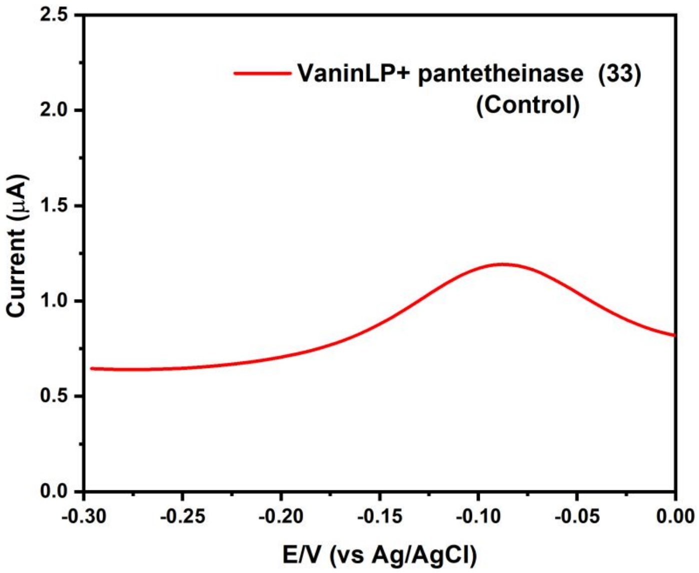
VaninLP+ pantetheinase (33)
(Control)
Current (μA)
E/V (vs Ag/AgCl)

Figure S11. DPV curves for VaninLP (50 μM) probe with different interfering species 1 to 30 1. Ascorbic acid, 2. Dopamine, 3. Uric acid, 4. L-cysteine, 5. Glutathione, 6. Glucose, 7. Creatine, 8. Urea, 9. Copper chloride, 10. Valine, 11. Phenylalanine, 12. Tryptophan, 13. Asparagine, 14. Threonine, 15. Sarcosine, 16. Calcium pantathienate, 17. Alanine, 18. Magnesium chloride, 19. Lysine, 20. Ferrous chloride, 21. Glycine, 22. Proline, 23. Arginine, 25. Calcium chloride, 26. GGT, 27. Esterase, 28. Leucine aminopeptidase, 29. H_2O_2, 30. Dipeptyl peptidase (DPP-IV), 31. Trypsin, 32. Aminopeptidase-N (APN), 33. Pantetheinase respectively.

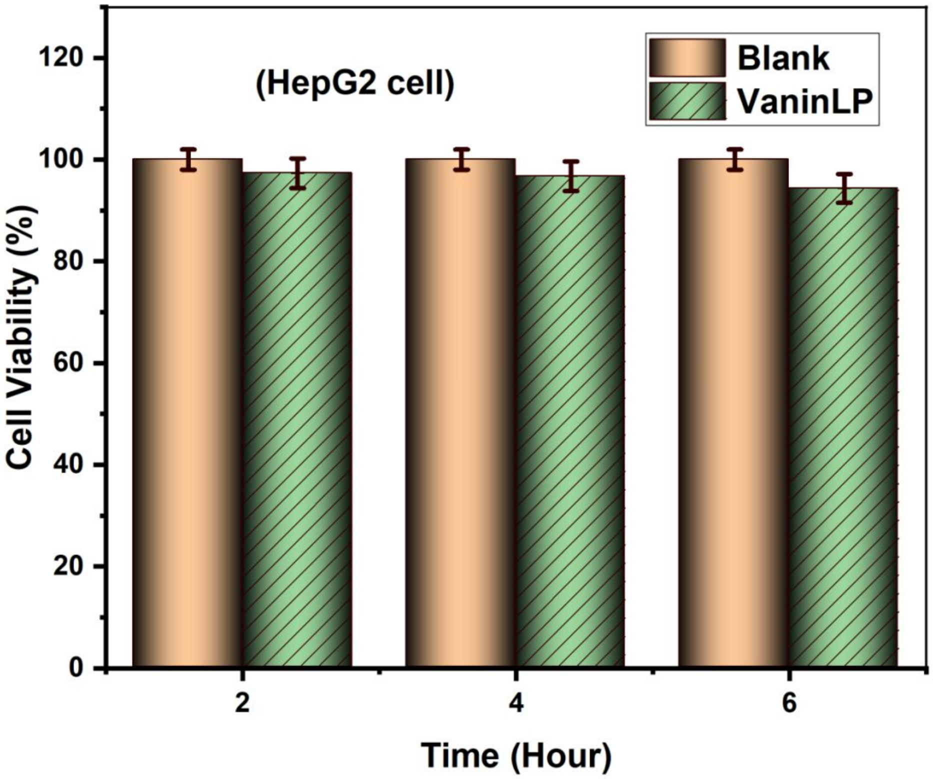

Figure S12. Bar chart of the cell viability (%) levels of HepG2 cell with and without VaninLP probe.

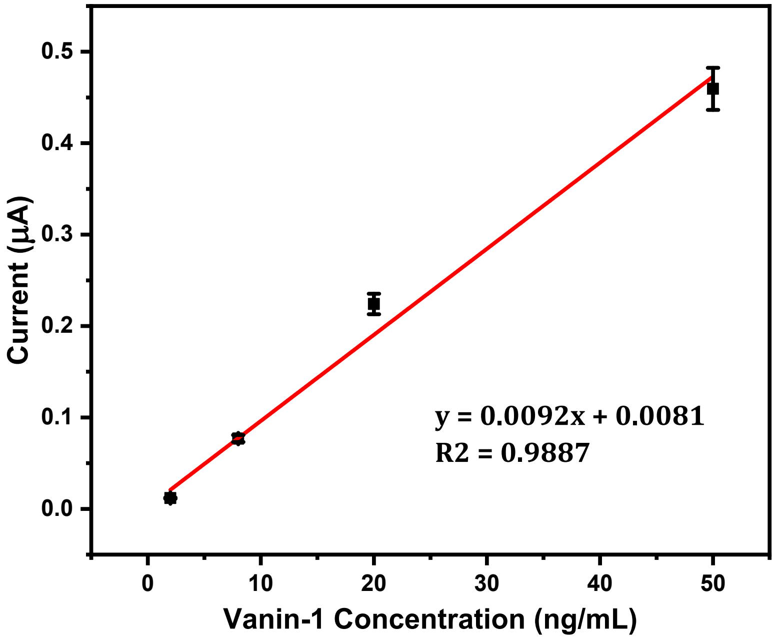

Figure S13. Calibration plot of current response against the concentration of pantetheinase, incubation for 5 hours. (Which is used for HepG2 cell quantification)

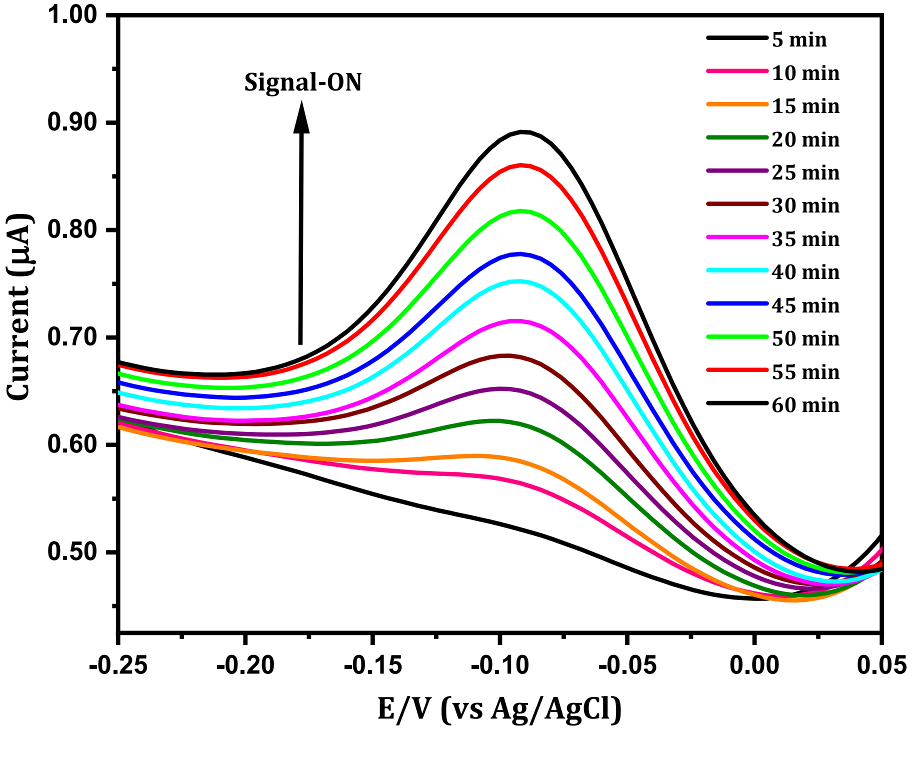

Figure S14. DPV figures of Real-time detection of NBCS without RR6.

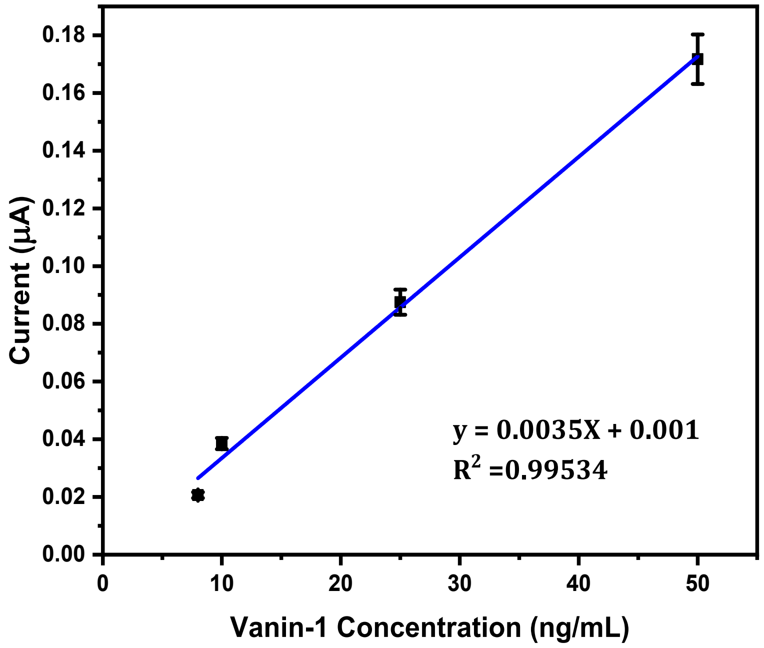

Figure S15. Calibration plot of current response against the concentration of pantetheinase, incubation for 30 mins (Which is used for urine pantetheinase quantification).

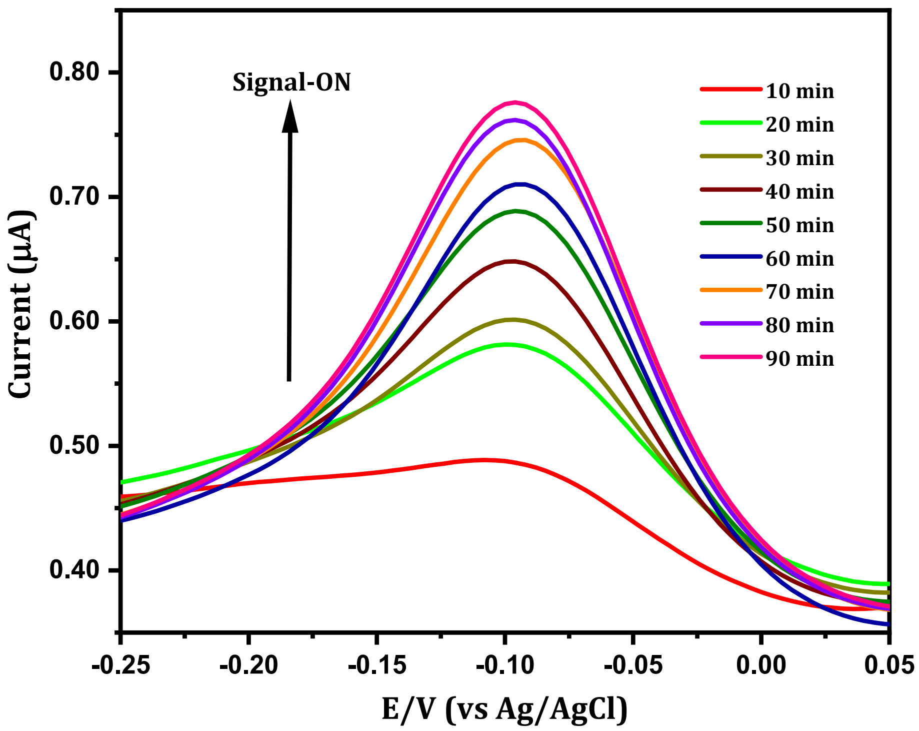

Figure S16. DPV figures of Real-time detection of blood without RR6.

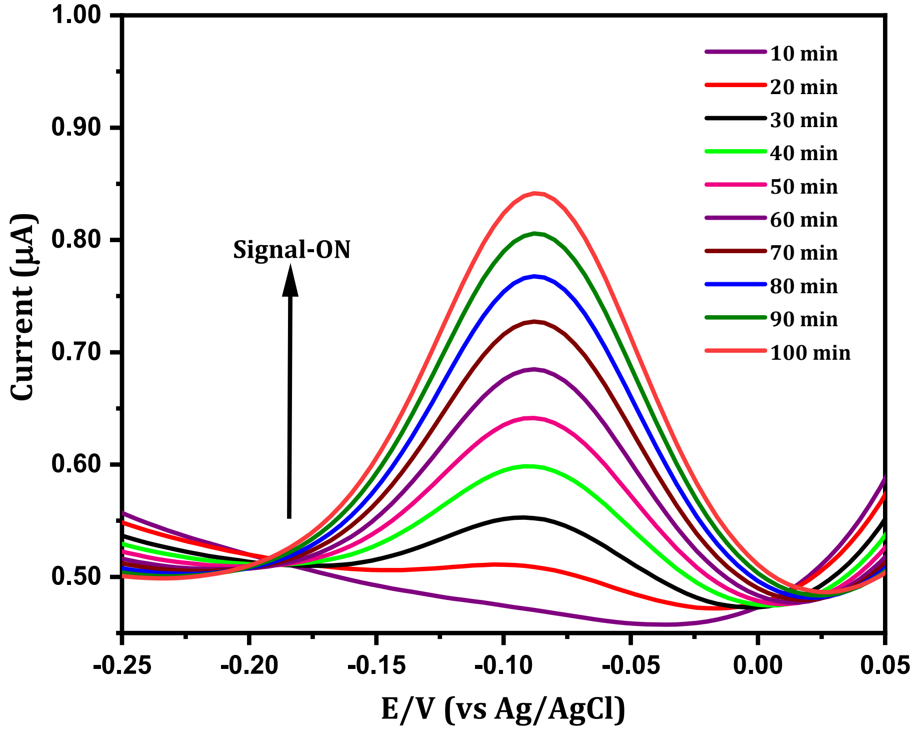

Figure S17. Corresponding DPV figures of Real-time detection of urine without RR6.

Reference:

(1) Hosohata, K.; Ando, H.; Fujiwara, Y.; Fujimura, A. Vanin-1; a Potential Biomarker for Nephrotoxicant-Induced Renal Injury, *Toxicology*, **2011**, *290(1)*, 82-88.

(2) Dupre, S.; Chiaraluce, R.; Nardini, M.; Cannella, C.; Ricci, G.; Cavallini, D. Continuous Spectrophotometric Assay of Pantetheinase Activity, *Anal. Biochem*., **1984**, *142(1)*, 175-81.

(3) Hu, Y.; Li, H.; Shi, W.; Ma, H. Ratiometric Fluorescent Probe for Imaging of Pantetheinase in Living Cells, *Anal chem.*, **2017**, *89(20)*, 11107-11112.

(4) Qian, J.; Teng, Z.; Wang, J.; Zhang, L.; Cao, T.; Zheng, L.; Cao, Y.; Qin, W.; Liu, Y.; Guo, H. Visible to Near-Infrared Emission Ratiometric Fluorescent Probe for the Detection of Vanin-1 In Vivo, *ACS sensors*, **2020**, *5(9)*, 2806-2813.

(5) Qian, J.; Zhang, L.; Wang, J.; Teng, Z.; Cao, T.; Zheng, L.; Cao, Y.; Qin, W.; Liu, Y.; Guo, H. Red Emission Ratio Fluorescent Probe for the Activity of Vanin-1 and Imaging in Vivo, *J. Hazard. Mater.*, **2021**, *401*, 123863.

(6) Yang, Y.; Hu, Y.; Shi, W.; Ma, H. A Near-Infrared Fluorescence Probe for Imaging of Pantetheinase in Cells and Mice in Vivo, *Chemical Science*, **2020**, *11(47)*, 12802-12806.

(7) Kumaragurubaran, N.; Tsai, H.T.; Arul, P.; Huang, S.T.; Lin, H.Y. Development of an Activity-Based Ratiometric Electrochemical Probe of the Tumor Biomarker γ-Glutamyl Transpeptidase: Rapid and Convenient Sensing in Whole Blood, Urine and Live-cell Samples, *Biosens Bioelectron.*, **2024**, *248*, 115996.
